# Low-amplitude copy number gains shape cancer through known and novel oncogenes with associated therapeutic vulnerabilities

**DOI:** 10.1093/nar/gkaf689

**Published:** 2025-07-22

**Authors:** Thomas F Eleveld, Bauke Ylstra, Leendert H J Looijenga

**Affiliations:** Princess Máxima Center for Pediatric Oncology, Heidelberglaan 25, 3584 CS Utrecht, The Netherlands; Amsterdam UMC, Vrije Universiteit Amsterdam, Department of Pathology, Cancer Center Amsterdam, 1081 BT Amsterdam, The Netherlands; Princess Máxima Center for Pediatric Oncology, Heidelberglaan 25, 3584 CS Utrecht, The Netherlands; Department of Pathology, University Medical Center Utrecht, 3584 CX Utrecht, The Netherlands

## Abstract

Large chromosomal copy number gains are ubiquitous throughout cancer types. However, which genes drive their selective advantage is not well established, and therefore they are hardly utilized in clinical practice. Our analysis of copy number patterns in pan-cancer datasets suggests that the selective advantage of copy number gains is largely driven by known oncogenes. Analysis of CRISPR screening data identifies a list of 101 genes that are likely to mediate the effect of these gains, which is highly enriched in annotated oncogenes but also contains genes that have not been implicated in cancer so far. Moreover, we show that specific gains are associated with drug sensitivity or resistance, with a strong enrichment of gains of oncogenes with increased sensitivity to inhibitors targeting these specific genes. Finally, we provide examples where gains can function as relevant clinical biomarkers for diagnosis and treatment. Thus, large copy number gains exert their selective advantage through known and novel oncogenes, and their systematic analysis could advance precision oncology.

## Graphical Abstract

**Graphical Abstract:**
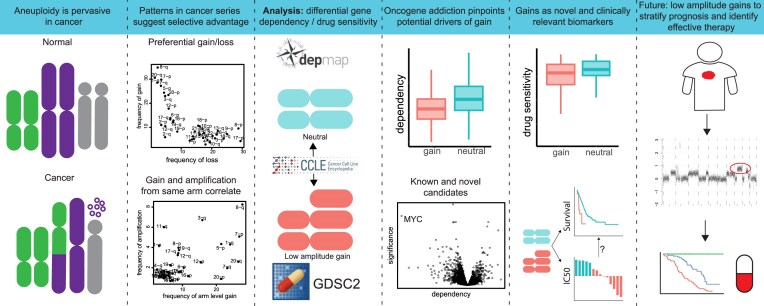

## Introduction

Somatic alterations in the DNA of cancer cells can be classified as single nucleotide variants, indels, and structural variants. Structural variants that affect the number of copies of a certain segment of DNA are known as copy number aberrations (CNAs). Whether and how specific somatic alterations contribute to cancer formation and/or progression can be relatively easily determined for single nucleotide variants, indels, and structural variants that do not affect chromosomal copy numbers by studying the genes or functional elements that are recurrently affected in large sample series. This has led to a wealth of knowledge on genes that when altered have unequivocal and established roles in cancer development and progression as well as (targeted) therapeutics that can be used to counteract their oncogenic effects [1]. For CNAs, this is more complicated because they affect larger regions of DNA, most often including multiple genes.

CNAs can be classified as focal CNAs, meaning affecting regions of limited size and thus including relatively few genes, and large CNAs, affecting whole chromosomes, chromosomal arms, or large regions thereof [2]. Gains and losses of whole chromosome arms or whole chromosomes are low-amplitude (involving a single copy for most cases), while focal gains can occur at both low and high amplitude [3]. High-amplitude losses are commonly known as deep deletions, meaning that all copies of a specific chromosomal region have been lost. High-amplitude focal gains are in classical cytogenetics referred to as amplifications occurring in solid cancers commonly as extrachromosomal DNA [4] that lacks centromeres. For many amplifications and deep deletions, the driving genes and associated pathways have been identified by delineation of the most common region of overlap between tumors in large datasets. For large low-amplitude CNAs, this has proven to be challenging due to the substantial number of genes simultaneously gained or lost.

Many large low-amplitude CNAs have been shown to be highly recurrent across several large pan-cancer series, with frequencies of the most common CNAs being similar to those of the most commonly mutated gene (*TP53*) and much higher than most other alterations [5]. About two-thirds of the CNAs involve gain or loss of entire chromosomal arms, and the other third, parts thereof [6]. The overall recurrence rates of whole-arm CNAs versus CNA events spanning less than a whole arm show that they likely occur through different mechanisms; however, the observation that they preferentially affect the same chromosomes suggests that they are both selected for by means of fitness advantage [6]. This implies that chromosomal aneuploidies at specific locations provide cancer cells with a selective advantage.

For recurrent chromosomal losses, several tumor suppressor genes have been resolved since they are either homozygously deleted or one of the hits in the two-hit hypothesis [7], though this is not the case for most recurrently affected chromosomal regions. Moreover, tumor suppressors can also be haplo-insufficient, suggesting that the driving events for a significant part of these aberrations remain to be determined. For large low-amplitude chromosomal gains, elucidation of driver genes is even more challenging, since the mechanism through which they would provide a selective advantage is less clear. Recent work has identified oncogenic drivers for some chromosomal regions that are frequently gained [8]; however, for the majority of regions of low-amplitude gain, the potential oncogenic drivers remain largely unknown.

To study the role of large low-amplitude CNAs, we investigated their frequency in large pan-cancer datasets and observed that specific chromosomal regions have a strong tendency for either gain or loss across cancer types. Moreover, the frequency of chromosomal arm gain correlates with the frequency of oncogene amplifications derived from the same arm, suggesting that these oncogenes also drive the selective advantage of these large low-amplitude gains, at least in part. This is supported by our observation that cancer cell lines with low-amplitude gain of oncogenes are more dependent on these oncogenes and more sensitive to specific inhibitors targeting these oncogenes. Finally, we provide multiple examples of large low-amplitude CNAs as novel biomarkers for prognosis and/or treatment, underlining their clinical potential.

## Materials and methods

### Data

Copy number segments, clinical data, and GISTIC calls (CNA genes) from the TCGA pan-cancer and MSKCC Impact datasets were downloaded from cBioPortal [9]. Data from the CCLE dataset were downloaded from the DepMap data portal (https://depmap.org/portal) [10]. Data from GDSC drug screening program were downloaded from the GDSC portal and data from the CTRP program were downloaded from the DepMap data portal. A list of all data used is shown in Supplementary Table S1.

### Analysis

**Diagram 1:**
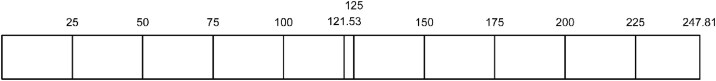
Schematic overview of the binning procedure for chromosome 1.

The copy number data used are the log_2_ ratios for each segment divided by the sample ploidy. Therefore, all gains and losses called are relative to the ploidy of the sample. Each chromosome was divided into bins of the specified size. The centromere region was added as an additional bin boundary to ensure that bins did not span the two arms of a chromosome. An example for chromosome 1 and 25-Mb bins is shown in Diagram 1.

**Diagram 2:**
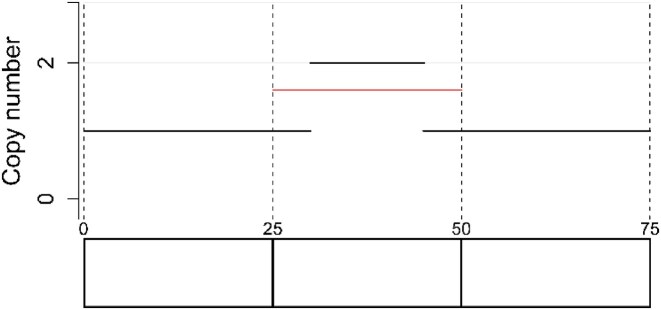
Example of the calculation of the CN for the bin 25–50 Mb. Segments in black are the actual segments and the segment in red is the calculated bin CN.

For each bin for each sample, the copy number is determined by selecting segments that fall within this bin and calculating the weighted mean segment copy number, with the percentage of the bin that shows the defined copy numbers as the weight (Diagram 2). Samples that show a copy number higher/lower than a predefined threshold per dataset are scored as gain/loss.

Bin sizes of 25 Mb were used for initial analyses to reduce noise and get a broad overview of large CNAs. Subsequently, bin sizes of 100 kb and 1 Mb (since 100 kb was not feasible in the elastic net regression) were used to pinpoint regions of interest. For gene-centered analyses, the gene start and end locations in the relevant genome build were used as bin boundaries.

*Z*-scores for gain and loss of specific bins were calculated as described earlier [2]. Arm-level gains were defined as chromosome arms where copy number was higher than a predefined threshold specific to the dataset used (Supplementary Table S1) for >80% of the length for which copy number data were available of that arm. High-level amplification status of genes in specific samples for the TCGA/MSKCC Impact was downloaded from cBioPortal. For the CCLE dataset, this information was incomplete, so genes were defined as showing a high-amplitude amplification if the copy number was higher than the highest copy number of a whole arm gain (as defined earlier) in the same sample.

For the associations between CRISPR/RNAi dependency and copy number gains, cell lines were classified as arm-level gain, focal gain, amplification, no gain, or loss for each gene. Subsequently, gene dependency scores were compared between cell lines with either low-amplitude gain (arm level and focal gain combined) or arm-level gain and cell lines with a neutral copy number by Student’s *t*-test, corrected for multiple testing by calculating the *q*-value where necessary using the qvalue R package. Genes that showed a combined importance of >0.05 for all experimental confounders in the best DepMap prediction model for that gene were removed from the analysis.

Gene set enrichment analysis (GSEA) was performed using the clusterProfiler package [11, 12]. Genes were ranked based on their *q*-value and analyzed for enrichment in the annotations provided by OncoKB [13] and the COSMIC Cancer Gene Census [14] as well as for a list of 50 most amplified genes in cancer [4].

For the associations between drug sensitivity and copy number gains, cell lines were classified as low-amplitude gain, amplification, or no gain for every gene. Subsequently, the area under the curve (AUC) for every compound was compared between cell lines with low-amplitude gain and cell lines without gain by Student’s *t*-test.

Elastic net regression was used for feature selection using the glmnet and caret R packages to model the AUC from (i) mutation status for 309 cancer genes as described in [15], (ii) copy number gain, loss, or amplification of 2827 1-Mb genomic bins according to the thresholds defined before, and (iii) cancer type and subtype of the included cell lines, as variables with five-fold cross-validation. The procedure was performed in triplicate for each dataset and only features that were included in all replicates are shown. Separately, all features were tested for their significance in an ANOVA analysis, including only the cancer type and subtype and that feature. *P*-values from this whole series were used for calculation of the *q*-value using the qvalue R package.

Associations between CNAs and patient survival were assessed using Kaplan–Meier analysis or Cox regression. For the figures using Cox regression analysis, the survival scores represent the hazard ratio for HR > 1, while they represents −1/HR for HR < 1.

### Software

Bioinformatic analysis was performed using R (version 4.4.0), R-studio (version 2023.03.1), and the R2 genomics analysis and visualization platform (R2.amc.nl).

List of R packages used including version:

**Table:** 

caret	6.0-94
clusterProfiler	4.12.5
corrplot	0.94
data.table	1.15.4
dplyr	1.1.4
enrichplot	1.24.2
ggplot2	3.5.1
ggpubr	0.6.0
glmnet	4.1-8
plyr	1.8.9
rstatix	0.7.2
survival	3.8-3
survminer	0.4.9
qvalue	2.36.0

### Code availability

Source code for all analyses can be found at Figshare https://doi.org/10.6084/m9.figshare.28956854.

## Results

### Preferential gain or loss of genomic regions throughout cancer types

**Figure 1.**
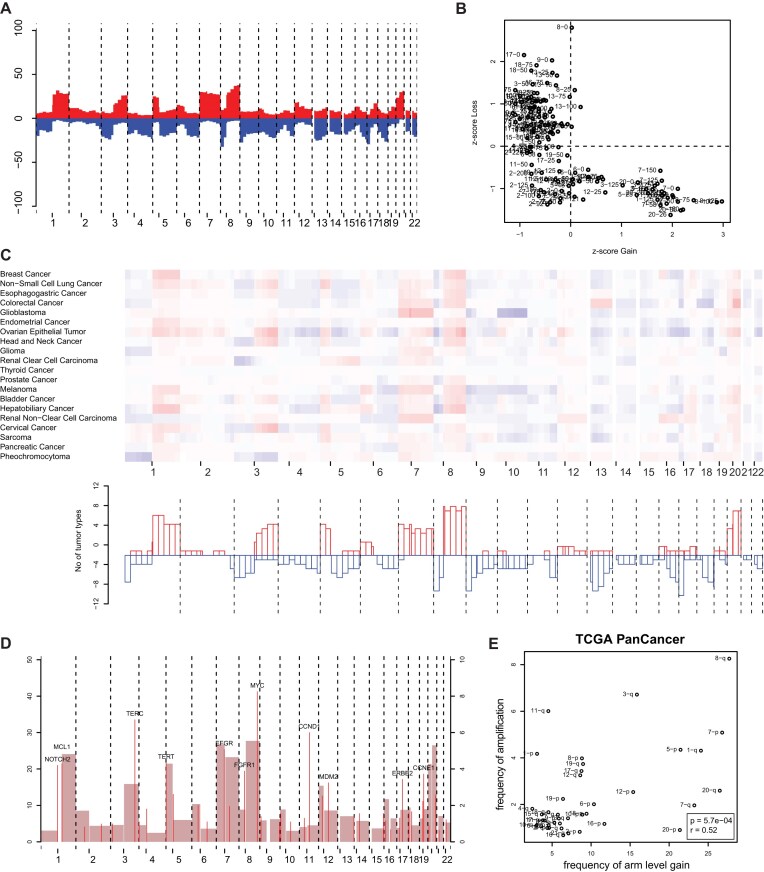
Large chromosomal CNAs are recurrent throughout solid cancer types and their frequency correlates with focal amplifications. (**A**) Frequency plot of chromosomal gains (positive) and losses (negative) in solid cancer from the pan-cancer TCGA dataset. On the *y*-axis the frequency of gain (positive) and loss (blue) is shown per genomic position represented by 25-Mb bins on the *x*-axis. (**B**) *Z*-scores for gain and loss of all bins shown in panel (A). (**C**) Tendency for gain (red) and loss (blue) within specific solid cancer types in the TCGA dataset. Upper graph: Colored bars represent the mean copy number per bin in the specified type. Lower graph: Bars represent the number of cancer types that show a frequency of >30% of gain (positive) or loss (negative). (**D**) Frequency plot of arm-level gains (dark, left *y*-axis) and the most frequent focally amplified genes per chromosome arm (bright vertical lines, right *y*-axis, only genes that are amplified in >3% of tumors are shown for readability). (**E**) *XY*plot showing the correlation between the arm level and focal gains per chromosome arm shown in panel (D).

To determine which large CNAs are most frequent throughout cancer types, the genome was divided into 142 25-Mb bins, and the weighted mean copy number for each bin was calculated using the segmented copy number data from pan-cancer datasets of TCGA and the MSK-Impact clinical sequencing cohort [16, 17]. For this analysis, the log_2_ ratio copy number was used, which is the metric that is directly generated by the diverse profiling methods and is agnostic to sample ploidy and does not require inference of copy numbers. As a consequence, all gains and losses that are defined represent gains or losses relative to the ploidy of the sample. Hematopoietic malignancies are known to show fewer large low-amplitude chromosomal copy number changes than solid cancers [18] and were excluded from these analyses, resulting in 10 516 and 10 764 tumor samples included in the analyses from the TCGA and MSKCC series, respectively. Subsequently, it was determined how frequently specific chromosomal bins were gained or lost compared to all other bins in the genome. This shows that specific chromosomal regions have a propensity for either loss or gain, and rarely show both frequent gain and loss, confirming that the occurrence and/or selection of these aberrations is not random (Fig. 1A and B, and Supplementary Fig. S1). Comparison of the TCGA and MSKCC datasets shows a very high concordance of values for each 25-Mb bin (*P*-value <2.2e−16, *R* value 0.87 and 0.87 for gain and loss, respectively, Supplementary Fig. S2), which means that the observed patterns in copy number gains and losses are very similar despite differences in sample series as well as CNA detection methods.

The observed tendency for gain or loss of a specific chromosomal bin appears largely independent of histological cancer type and origin, with bins showing a propensity for either gain or loss across multiple cancer types (Fig. 1C and Supplementary Fig. S3). There are some notable exceptions, like gain of chromosome 13 in colorectal cancer, which is lost in most other cancer types, likely due to expression of specific genes on this chromosome that is indispensable for this tissue type [19]. The fact that chromosomal regions are either unaffected or preferentially gained or lost, and that the cancer origin and/or type is not of major influence on this preference, suggests that most recurrent chromosomal CNAs provide a general selective advantage in tumor formation and/or progression that overarches subtype.

### Low-amplitude gain of oncogenes has an oncogenic effect

To obtain more insight into the oncogenic function of large low-amplitude chromosomal gains, we determined whether amplifications and whole chromosome arm gains affect the same genomic regions. This comparison showed a significant correlation between the frequency of low-amplitude gain of whole chromosome arms and the frequency of amplifications involving the same chromosomal arm for both the TCGA and the MSKCC impact datasets (Fig. 1D and E, and Supplementary Fig. S4). Also, within cancer types there is a significant correlation between arm-level large gain and focal amplification in 11/15 cancer types in the TCGA and 9/15 cancer types in the MSKCC set (Supplementary Fig. S5).

This resulted in the hypothesis that low-amplitude gain of whole chromosome arms and high-amplitude amplifications exert a selective advantage, at least in part, through the same oncogenic mechanisms. To test this hypothesis, rather than looking into patient data, we analyzed cancer cell lines in the DepMap dataset, where 881 cell lines originating from solid cancers were subjected to CRISPR screening, determining how dependent each cell line is on each specific gene [20]. The copy number pattern of these cell lines closely resembles that of the pan-cancer datasets described earlier (Supplementary Fig. S6), indicating that it is a representative dataset for this purpose. It has been established that oncogenic mutations are associated with increased dependence on the involved genes [10], which prompted us to determine differential dependencies between cell lines that show gain of specific genomic regions versus those cell lines with a neutral copy number.

**Figure 2.**
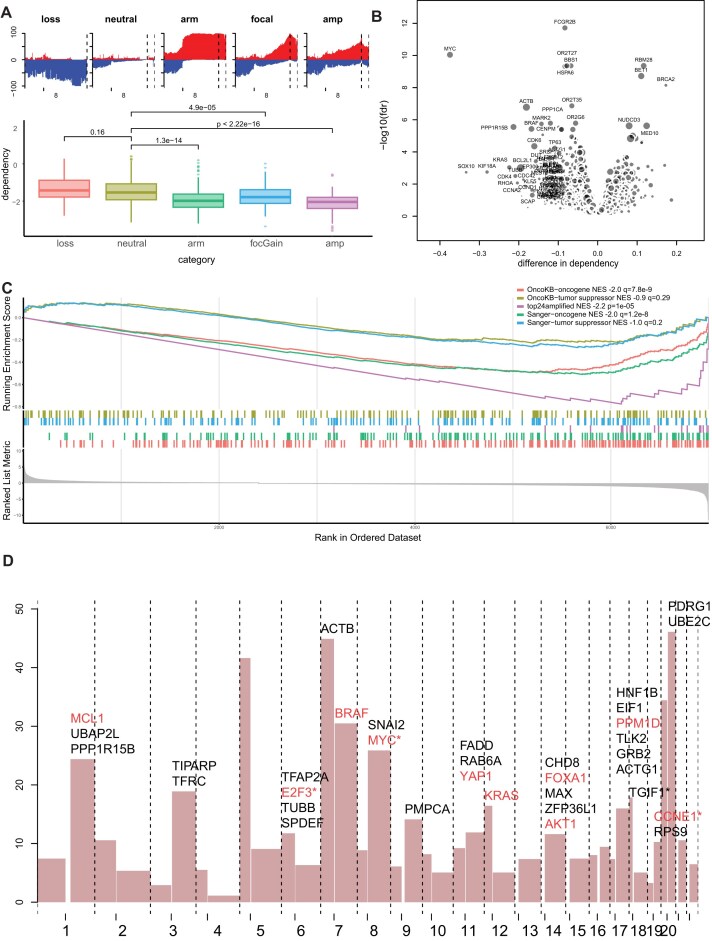
Low-amplitude copy number gains are associated with increased oncogenic dependencies. (**A**) Boxplot showing dependency on MYC in cancer cell lines with loss of MYC, with neutral MYC copy number, with gain of the whole q arm of chromosome 8, with focal low-level gain of MYC, and with amplification of MYC. Frequency plots show the frequency of gain (positive) and loss (negative) of chromosome 8 bins in these cell lines. (**B**) Volcano plot showing differential CRISPR gene dependency based on copy number status of the gene. Dot size is relative to the frequency of gain of this region. Genes on the left side of the graph show higher dependency in cell lines with copy number gain. (**C**) GSEA of the analysis depicted in panel (B). Genes are ordered based on the log_10_(*q*-value), with the sign determined by the dependency difference. Curves represent running enrichment scores of the indicated gene sets in the ranked data. Colored bars represent the position of the genes in the respective sets in the ranked list. (**D**) Frequency plot of arm-level gains. Shown above chromosome arms that have a frequency of gain >10% are the genes that show significant differential dependency when gained. Genes annotated in OncoKB as oncogenes are colored red and the most frequently gained gene on each arm is marked with an asterisk. There are 14 additional genes in the 8-Mb region preceding the FADD gene on chromosome 11q (TAF6L, MARK2, CDC42EP2, SCYL1, MAP3K11, RELA, FOSL1, RAB1B, NPAS4, BBS1, GRK2, PPP1CA, CHKA, and PPP6R3) but these were left out of the graph for readability.

The long arm of chromosome 8 (i.e. 8q), specifically the bin from 125 Mb to the telomeric end, is the region that is most frequently gained in both pan-cancer datasets described earlier and the DepMap cell lines. To determine whether this leads to increased dependency of genes located in this region, cell lines with amplification of the *MYC* gene (the most frequently amplified gene on this arm, Fig. 1D and Supplementary Fig. S4) were compared with cell lines with (i) gain of the whole 8q arm, (ii) low-amplitude focal gain spanning the *MYC* gene, (iii) no gain, and (iv) loss of the MYC region (Fig. 2A). Cell lines with amplification of this region show a significant (*P*= 3.6e−24, difference in means = −0.64) differential dependency on the *MYC* gene compared to cells without gain of the *MYC* region. In concordance with the hypothesis that low-amplitude gain of *MYC* also gives an oncogenic advantage, we observe significantly increased dependency in cells with gain of the whole arm (*P*= 1.3e−14, difference= −0.44) and low-amplitude focal gain (*P*= 4.9e−05, difference= −0.28) of *MYC*, compared to cell lines with neutral copy numbers of *MYC*. Within the different cancer types represented in the dataset, gain of *MYC* is associated with high MYC dependency without exception, while cell lines without gain are significantly less dependent in three cancer types (bladder cancer, bone cancer, and ovarian cancer), while there is no difference in the remaining 10 types (Supplementary Fig. S7). This suggests that in the latter cancer types there could also be other aberrations that contribute to *MYC* dependency, such as *KRAS* or *EGFR* mutations that have been shown to increase MYC activation [21, 22].

### Genome-wide analysis of oncogenic drivers through low-level copy number gain

To determine whether low-amplitude copy number gain also associates with increased dependency for genes in other genomic regions, a genome-wide analysis was performed, in which all low-level gains (whole chromosome arm and focal) were combined in one group to maximize the power of detection. For each gene, it was determined whether there was a differential gene dependency between cancer cell lines with low-amplitude gain spanning the gene and cell lines with a neutral copy number (Fig. 2B). Interestingly, genes that showed higher dependency when gained were significantly enriched (NES = −2.2, *P*= 1e−0.5) for the 24 most commonly amplified genes in cancer [4], indicating that also low-level gain of commonly amplified genes causes increased dependency (Fig. 2C). To ascertain whether this pattern (increased dependency with low-level copy number gain) is more broadly observed for oncogenes, enrichment analysis was performed based on the annotation of genes involved in cancer in OncoKB [23] and the COSMIC Cancer Gene Census [14]. Enrichment of oncogenes is observed in genes showing this pattern, while this enrichment was not observed for genes that are annotated as tumor suppressor genes (Fig. 2C). This finding was validated in a large RNAi screening dataset [24] and an independent CRISPR screening dataset [25], in which we observed the same patterns (Supplementary Fig. S8). This analysis confirms that genes that show increased dependency when gained show an enrichment of oncogenes.

To compile a systematic list of genes that could be important for the driving effect of low-amplitude chromosomal gains in oncogenesis, we selected the candidates that showed a significantly lower dependency score, meaning that they are more dependent on this gene (False Discovery Rate (FDR) *q*-value <0.3, difference in dependency <−0.05) in cell lines with low-amplitude gain compared to cell lines with a neutral copy number in at least two of the three aforementioned datasets. The most overlap was observed between the DepMap and Sanger datasets (86 genes overlapping), which were both generated using CRISPR screening, while the overlap with the RNAi screening dataset was less (22 and 9 overlapping with DepMap and Sanger, respectively, Supplementary Fig. S8). This yielded a list of 101 genes (Supplementary Table S2 and Fig. 2D). Of these 101 genes, 21 are listed as oncogenes by OncoKB and 14 (all overlap with OncoKB) in the COSMIC Cancer Gene Census (*P*= 4.6e−14 and 9.5e−07, respectively, by chi-square test). Eight genes are identified in all datasets (*ACSL3*, *CCNE1*, *CDK4*, *PDGFRA*, *PPP1CA*, *PP1R15B*, *RAB6A*, and *TEAD1*), of which three (*CCNE1*,*CDK4*, and *PDGFRA*) are established oncogenes. For most frequently gained chromosome arms, the list of 101 genes identifies only a small number of genes, e.g. only 2 for the 8q arm, *MYC* and *SNAI2*, with *MYC* showing a significantly higher effect (dependency difference −0.39 *q*-value 5.2e−12 for *MYC* versus −0.07 and 0.01 for *SNAI2*). An exception is chromosome 11q, which shows a cluster of 16 genes within a region of 11 Mb that show this pattern (Supplementary Fig. S9 and Supplementary Table S2).

Moreover, we analyzed for each chromosomal arm which 100-kb bin region was most frequently gained in the TCGA solid cancer dataset, since the most common region of overlap is expected to center on the genes that are most beneficial for cell growth (Supplementary Fig. S10). Interestingly, for six chromosomal arms, a most frequently gained bin overlapped with a gene in our candidate list (marked by an asterisk in Fig. 2C), while six more frequently gained genes (*SLBP*,*EGFR*,*CDK6*,*TUBB4B*,*CCND1*, and *WNK1*) were identified as significant (*q*-value <0.3, difference in dependency <−0.05) in one of the three analyzed datasets. These in total 12 genes contain 8 oncogenes as defined by the OncoKB database (*PDGFRA*, *E2F3*, *EGFR*,*CDK6*,*MYC*, *CCND1*, *IGF1R*, and *CCNE1*) but also 4 genes with a lesser known role in cancer (*SLBP*,*WNK1*, *TGIF1*, and *TUBB4B*), which suggests that these genes might be important in oncogenesis through low-amplitude copy number gain. In these analyses, all low-amplitude gains were compared with cell lines with neutral copy numbers, yet when analyzed only with cell lines with whole-arm gains versus no gain, results show a similar enrichment of oncogenes with 65 of the list of 101 (64%) genes also showing increased dependence when the whole arm is gained (Supplementary Fig. S11 and Supplementary Table S2). This suggests that low-amplitude focal gain and whole chromosomal arm-level gains exert a similar oncogenic function through the activation of known and novel oncogenes.

Of the 101 genes in the list, the vast majority showed significant upregulation of gene expression in samples with low-amplitude gain compared to samples with neutral copy number in both the TCGA (92/101 upregulated, 6/101 not upregulated, and 3/103 unevaluable genes) and the CCLE datasets (84/101 upregulated, 14/101 not upregulated, and 3 unevaluable genes) (Supplementary Table S2).

To determine whether there is an interaction between mutations in these 101 genes and low-amplitude gain, we also analyzed their co-occurrence in the CCLE and TCGA datasets. Low-level gains and mutations co-occurred significantly more frequently than expected for KRAS (gain in 24% of mutated tumors versus 18% in non-mutated tumors, *q* = 0.02) in the TCGA dataset, as reported before in lung cancer [26], and KRAS (gain in 40% of mutated cel lines versus 23% in non-mutated cell lines, *q* = 1.0e−03) and BRAF (gain in 65% of mutated cell lines versus 40% in non-mutated cell lines, *q* = 5.0–06) in the CCLE dataset, while no mutually exclusive patterns were detected. This suggests that gain of these specific chromosomal regions could benefit cancer cell growth by increasing the copy number of mutated genes. In line with this, we see a significant difference in dependency on *BRAF* and *KRAS* based on their copy number status in cell lines with hotspot mutations for these genes. A significant difference in dependency based on copy number status was also observed in the non-mutated cell lines for *KRAS*, but not for *BRAF* (Supplementary Fig. S12), suggesting that for *KRAS* also increasing the non-mutated copy number is beneficial for the cancer cells.

### Low-amplitude chromosomal gains are associated with therapeutic vulnerabilities

**Figure 3.**
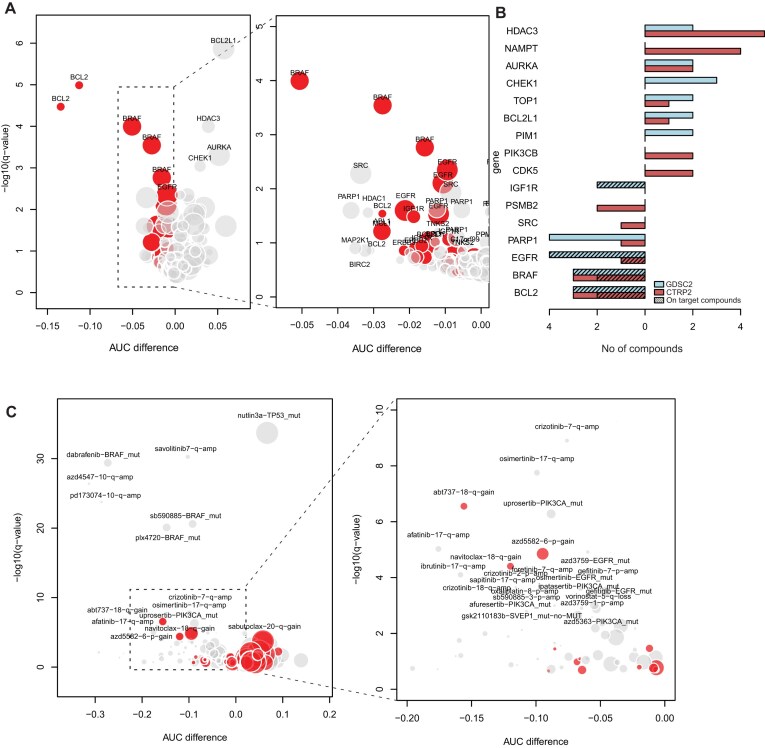
Low-amplitude copy number gains are associated with sensitivity and resistance to specific compounds. (**A**) Left: Volcano plot showing on-target combinations in the GDSC2 dataset. Every dot represents a drug–gene combination (indicated here by only the gene name for clarity). Dot size is relative to the frequency of gain of this region. Dots to the left side of the pattern represent compounds that show increased sensitivity when the target gene is gained. Red dots represent on-target compounds. Right: Zoomed in view of the graph area in the dashed box in the left graph. (**B**) Barplot showing the number of compounds that show sensitivity (left) or resistance (right) when their target gene is gained. Colors represent the dataset and shading indicates on-target compounds (compounds that show a strong correlation with target gene dependency). (**C**) Left: Volcano plot showing feature–compound associations identified by elastic net regression in both the GDSC2 and CTRP2 datasets. Dot size is relative to the frequency of gain of this region. Red dots represent associations involving low-amplitude gains. Right: Zoomed in view of the graph area in the dashed box in the left graph.

Subsequently, we hypothesized that the low-amplitude gains of oncogenes could also be associated with sensitivity to treatment with compounds targeting these oncogenes, as has been observed for amplifications and mutations [15]. To analyze this, we queried the Cancer Genomics of Drugs Sensitivity 2 (GDSC2, 286 drugs tested in 770 cancer cell lines) [27] and Cancer Therapeutics Response Portal (CTRP, 545 compounds or combinations tested in 675 cell lines) [28] datasets, which contain a large degree of overlap in cell lines with the previously used DepMap dataset. We selected compounds with a known target gene and determined whether increased copy number of these target genes was associated with increased sensitivity or resistance to the compound under study. In total, 52/316 compounds show a significant (*q*-value <0.1) difference in AUC when comparing cell lines with versus without a gain in their target gene, with 23 compounds showing significantly increased sensitivity in cell lines with gain and 29 compounds showing increased resistance in cell lines with gain (Fig. 3A and Supplementary Table S3). When only considering compounds that show a pattern where increased dependence on the gene is associated with increased sensitivity (*r* > 0.3 and *P*-value <1e−10 using Pearson correlation, referred to as “on-target compounds” from here), as would be expected for classical oncogene inhibitors, 14/40 of these compounds show significantly increased sensitivity when their target gene is gained, while 1/40 shows increased resistance when their target gene is gained. For the CTRP2 datasets, the results of this analysis are similar, with 14 compounds showing sensitivity when their target gene is gained versus 23 showing resistance when their target gene is gained. For the on-target compounds, 5/34 show increased sensitivity when their target gene is gained, while none show increased resistance (Supplementary Fig. S13 and Supplementary Table S4). In aggregate, BRAF, BCL2, and EGFR inhibitors, which are mostly on-target compounds, most frequently show increased sensitivity when their target gene is gained, while HDAC3, NAMPT, and AURKA inhibitors most frequently show resistance when their target gene is gained (Fig. 3B). This shows that low-amplitude gain of oncogenes can have a considerable influence on sensitivity to inhibitors targeting those genes.

To determine whether copy number gains can be useful biomarkers for drug sensitivity in the framework of other existing aberrations in these cell lines, elastic net regression was performed to model AUC for all drugs using (i) mutation status for 309 cancer genes as described in [15], (ii) copy number gain, loss, or amplification of 2827 1-Mb genomic bins, and (iii) cancer type and subtype of the included cell lines, as variables. This approach selects the variables that show the strongest independent association with drug response. The strongest associations are with cancer type or subtype for most compounds (138/295 compounds in the GDSC2 and 238/496 in the CTRP2); however, these were left out of subsequent analyses for clarity. Of the remaining variables, the strongest associations are observed for mutations and amplifications. These involve, among others, *BRAF* mutations associated with sensitivity to BRAF inhibitors and *MET* and *FGFR2* amplification and sensitivity to their respective inhibitors, confirming the validity of the used methodology (Fig. 3C).

Of the 139 genetic features associated with drug resistance or sensitivity in both the GDSC2 and CTRP2 datasets, 32 were low-level gains (Supplementary Table S5). They are associated with both sensitivity (13/32) and resistance (19/32), while mutations (20/22) and amplifications (33/33) were mostly associated with sensitivity. Seven out of 33 of the low-amplitude gains showed (partial) overlap with the Recurrently Aberrant Copy number Segments (RACS) in the GDSC2 analysis [15]. Only two out of these seven were also identified as significant there, while 21/22 of the associations for mutations in the list were also identified as significant in the GDSC2 analysis. This discrepancy could be due to the difference in methodology or the cancer types included in the analysis.

Some associations for low-level gains, such as 18q gain and sensitivity to BCL2 inhibition and 6p gain and sensitivity to IAP inhibition, were similar in strength to established biomarker–drug relationships such as *PIK3CA* mutations and PI3K/AKT inhibitor sensitivity and *EGFR* mutations and EGFR inhibitor sensitivity. This underlines the potential of identifying novel powerful biomarkers by systematically analyzing low-amplitude CNAs. Besides these general conclusions, our classification also yielded several novel associations that will be elaborated upon below.

### Low-amplitude gain of chromosome 6p25 is associated with sensitivity to IAP inhibition

**Figure 4.**
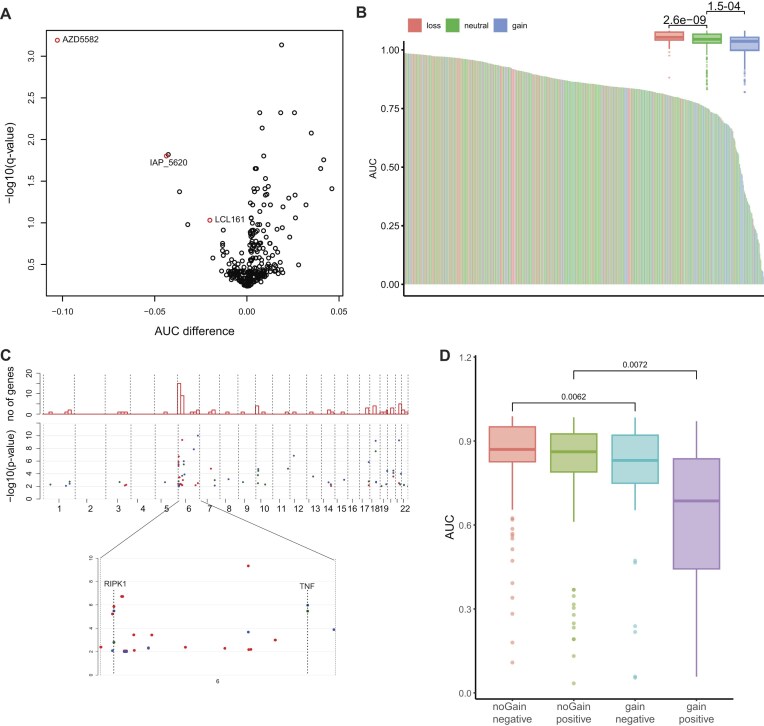
6p25 (3–4 Mb) gain as a specific biomarker for IAP inhibitor sensitivity. (**A**) Volcano plot showing associations between gain of chromosome 6p25 (3–4 Mb) and compound sensitivity. Every dot represents one compound and IAP inhibitors are colored red. Dots in the upper left part of the graph represent compounds that show increased sensitivity in cell lines with gain of this region. (**B**) Waterfall plot showing compound sensitivity for AZD5582 [see panel (A)]. Red represents cell lines with loss, blue cell lines with neutral copy number, and blue cell lines with low-amplitude gain. The boxplot in the upper right shows a comparison of the same groups; *P*-values were generated by *t*-tests. (**C**) Genes correlated with IAP inhibitor sensitivity and their chromosomal location. Red dots are for AZD5582, green dots for LCL161, and blue dots for IAP_5620. The upper bars represent the total number of genes per 25-Mb chromosomal bin. The genes RIPK1 and TNF are indicated. (**D**) Boxplot showing AZD5582 sensitivity in cell lines based on the expression of the three-gene signature proposed by Bardia *et al.* [32] combined with 6p25 (3–4 Mb) gain status. *P*-values were generated using *t*-test.

One of the strongest associations identified for low-amplitude gains with drug response was between gain of chromosome 6p25 (3–4 Mb) and sensitivity to the IAP (inhibitor of apoptosis) inhibitor AZD5582 in the GDSC2 dataset (*P*-value 1.7e−7, AUC difference of 0.11, Figs 3A and 4A and B). The IAP family of proteins is frequently activated in cancer, where it inhibits programmed cell death. Inhibitors of these proteins can, therefore, sensitize cancer cells to apoptosis [29]. Significant associations were also detected between low-amplitude gain in this region and other IAP inhibitors in the GDSC2 dataset (Fig. 4A). Sensitivity to the IAP inhibitors birinapant (*P*-value 5.2e−06, AUC difference −5.3) and AT-406 (*P*-value 8.8e−04, AUC difference −3.0) also showed a significant association with gain of 6p25 (3–4 Mb) in the CTRP2 dataset, as did all four IAP inhibitors from the GDSC1 dataset (Supplementary Fig. S14) [30]. To identify genes that could play a role in the observed sensitivity, compound sensitivity was subsequently correlated to gene expression for the compounds identified in the GDSC2 dataset. Interestingly, there is a strong enrichment of genes located on the p-arm of chromosome 6 in the genes that show a significant correlation with drug sensitivity (Fig. 4C, *P*< 2.2e−16 for AZD5582), with the top 4 most strongly correlated genes (*TNF*, *CDYL*, *RIPK1*, and *WRNIP1*) all being located in this region. Moreover, on protein level we also observe an enrichment of proteins from which the associated genes are located on chromosome 6p25 in correlated proteins, including the aforementioned CDYL, RIPK1, and WRNIP1 proteins (Supplementary Fig. S15) [31]. Two of these genes (*RIPK1* and *TNF*) were recently reported as part of a three-gene signature that identifies breast cancer patients that benefit from the IAP inhibitor LCL161, administered in combination with paclitaxel [32]. Sensitivity scores generated through pairwise mRNA expression ratios of these three genes as described in [32] show an association with sensitivity to all IAP inhibitors (Supplementary Fig. S15). Interestingly, using both gene expression and 6p25 (3–4 Mb) status leads to better classification of sensitive cell lines (Fig. 4D and Supplementary Fig. S15), indicating that it could be a useful clinical biomarker for stratification of patients for IAP inhibitor treatment. Overall, this suggests that low-amplitude copy number gain of chromosome 6p causes sensitivity to IAP inhibitor treatment through increased expression of *RIPK1* and *TNF*.

### Low-amplitude gain of chromosome 1q21 is associated with poor prognosis and MCL1 inhibitor sensitivity in neuroblastoma

**Figure 5.**
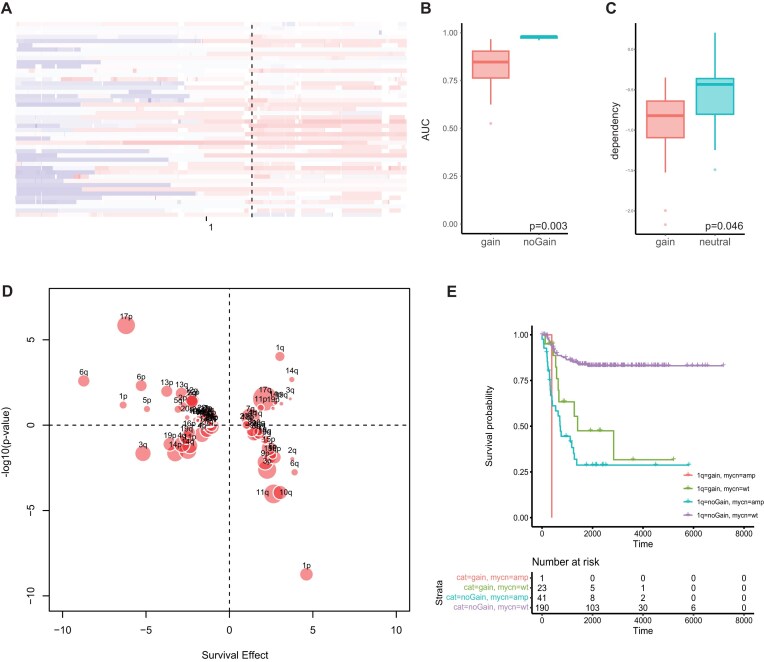
Low-amplitude gain of MCL1 as a biomarker for MCL1 inhibitor sensitivity and poor prognosis in neuroblastoma. (**A**) Copy number profiles of chromosome 1 in neuroblastoma cell lines with red depicting gain and blue depicting loss. (**B**) Boxplot showing AZD5991 sensitivity in neuroblastoma cell lines with and without MCL1 copy number gain. (**C**) CRISPR dependency on MCL1 in neuroblastoma cell lines with MCL1 copy number gain and with neutral copy number. (**D**) Bubble plot showing the association between copy number loss (negative *y*-values) or gain (positive *y*-values) and survival of neuroblastoma patients (positive *x*-values signify poorer survival and negative *x*-values signify better survival). (**E**) Kaplan–Meier curve showing survival of patients depending on MYCN amplification status and presence of MCL1 gain.

Another relationship identified was between low-amplitude gain of *MCL1* and sensitivity to MCL1 inhibitors (Fig. 3B). MCL1 is an anti-apoptotic protein from the BCL2 family and the corresponding gene is located on chromosome 1q21 [33]. We observed this association across cancer cell lines within the GDSC2 dataset across tumor types, and a similar tendency was also found within cancer types, with the strongest association for neuroblastoma cell lines (Fig. 5A and B). Moreover, neuroblastoma cell lines with 1q21 gain are significantly more dependent on *MCL1* compared to cell lines without in the DepMap CRISPR screening dataset (Fig. 5C). To evaluate the role of CNAs in survival of neuroblastoma patients and whether activation of MCL1 through 1q21 gain also plays a role in neuroblastoma patient prognosis, the association between the presence of gains and losses and survival was determined using Cox regression in two independent datasets [34, 35]. This analysis yielded established associations between chromosomal CNAs and neuroblastoma patient survival, like loss of chromosomes 1p and 6q [36, 37], validating our approach. Although 1q gain is less well established as a marker of poor prognosis in neuroblastoma, it also shows a significant association with poor patient survival in both analyzed datasets (Fig. 5C and Supplementary Fig. S16). Moreover, in the largest dataset, gain of the *MCL1* region on 1q21 is mostly mutually exclusive with *MYCN* amplification (chromosome 2p24), although this was not observed in the smaller dataset (Fig. 5D and Supplementary Fig. S16). This suggests that increased *MCL1* activity through low-amplitude copy number gain in neuroblastoma may contribute to an aggressive phenotype in tumors without *MYCN* amplification, which is the strongest predictor of poor prognosis in neuroblastoma tumors [38]. MCL1 inhibition might also present a potential therapeutic option for tumors with this CNA.

### Low-amplitude gain of chromosome 3p25 is associated with cisplatin resistance

**Figure 6.**
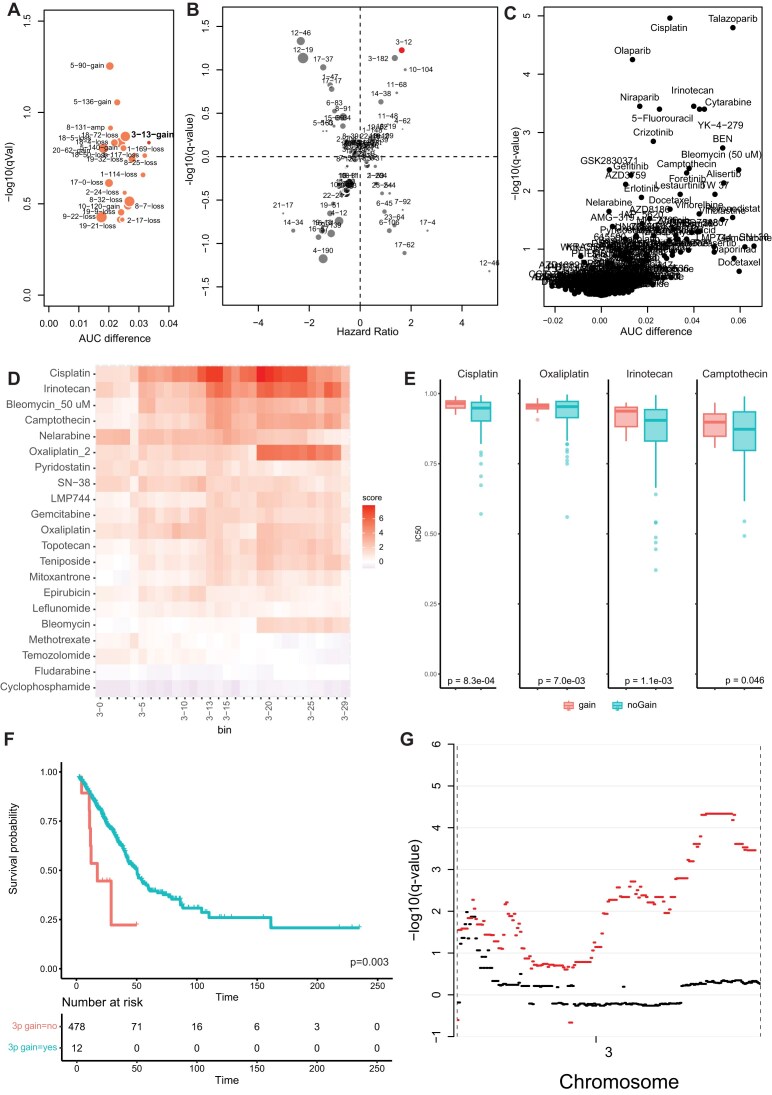
Low-amplitude gain of chromosome 3p25 (13–14 Mb) as a biomarker of cisplatin sensitivity. (**A**) Bubble plot showing AUC difference and *p*-value of the features identified in the elastic net regression modeling of cisplatin sensitivity in the GDSC2 dataset. Circle size represents the frequency at which features occur. Only features associated with resistance are shown. (**B**) Bubble plot showing the association between copy number loss (negative *y*-values) or gain (positive *y*-values) and survival of testicular malignant germ cell tumor patients (positive *x*-values signify poorer survival and negative *x*-values signify poorer survival). The size of the circles represents the frequency at which the feature occurs in the tumor series. (**C**) Volcano plot showing associations between gain of chromosome 3p25 (13–14 Mb) and compound sensitivity. Dots in the upper right represent compounds that show increased resistance in cell lines with gain of this region. (**D**) Heatmap showing association between gain of bins 0–30 Mb on the p arm of chromosome 3 and compounds affecting DNA replication. Colors represent the −log_10_(*P*-value) of the association between gain of this region and resistance (red) or sensitivity (blue) to the depicted compounds. (**E**) Boxplots showing the sensitivity to the indicated compounds based on 3p gain status in lung cancer cell lines. (**F**) Kaplan–Meier curve showing survival of lung adenocarcinoma patients in the TCGA depending on 3p gain status. (**G**) Significance of survival associations between gain of the indicated regions on chromosome 3 and poorer (positive *y*-values) or better (negative *y*-values) in various lung adenocarcinoma datasets (black = TCGA, red = MSKCC Impact).

Finally, we focused on the role of low-amplitude copy number gains in cisplatin resistance. In the previously mentioned elastic net regression analysis of the GDSC2 dataset, we identified gain of chromosome 3p25 (13–14 Mb) as the region of gain most strongly associated with cisplatin resistance across cancer types (Fig. 6A). Cisplatin is a widely used chemotherapeutic that has shown to be particularly effective for the treatment of patients with malignant (testicular) germ cell tumors [39]. Interestingly, we have recently described gain of chromosome 3p25.3, directly adjacent to the region identified in the cell line panel, in these cancers as a marker of resistance [40]. Gain of this region is indeed the strongest biomarker of poor response in malignant non-seminomatous germ cell tumors (*P*-value 2.7e−04, HR 2.6, Fig. 6B) [41], suggesting that gain of this chromosomal region could be relevant for cisplatin resistance across cancer types. Besides cisplatin, gain of this region is also associated with resistance to multiple other compounds with similar mechanisms of action, such as irinotecan and camptothecin, in the GDSC2 drug dataset (Fig. 6C). Further analysis shows a relationship between the copy number of bins in this region and resistance to several compounds affecting DNA replication (Fig. 6D), suggesting that gain of 3p25 is associated with a general resistance to DNA-damaging compounds. The relationship between chromosome 3p25 (13–14 Mb) gain and cisplatin was not detected in the CTRP2 dataset; however, several other DNA-damaging compounds (bleomycin, gemcitabine, irinotecan, and cytarabine) were among the top compounds showing resistance if this region is gained. In the GDSC1 dataset, cell lines with 3p25 gain (13–14 Mb) also showed higher resistance than cell lines without, although this was not significant for one of the two independent entries of cisplatin in this dataset (Supplementary Fig. S17).

The association between 3p25 gain (13–14 Mb) and resistance to DNA-damaging compounds was also observed when the same analysis was performed within cancer types in the CCLE panel. The highest significance was seen for lung cancer, where cell lines with gain were significantly more resistant to several other DNA-damaging compounds than cell lines without (Fig. 6E). Since cisplatin is used in the treatment of the vast majority of patients with lung cancers [42], gain of this region would also be expected to be associated with poor outcome if it is involved in cisplatin resistance. Gain of 3p25 (13–14 Mb) was indeed significantly associated with poor survival in patient series of lung adenocarcinomas in the TCGA and MSKCC Impact datasets (Fig. 6F and Supplementary Fig. S18). Analysis of all bins on chromosome 3 shows that the specific region that is associated with cisplatin resistance in cell lines is in the same region that is most significantly associated with poor survival on chromosome 3p in the patient series (Fig. 6G). A significant part of the MSKCC cohort was also reported to have been treated with immune checkpoint inhibitors [43]. Interestingly, although the numbers are low, patients with 3p25 (13–14 Mb) gain treated with immune checkpoint inhibitors did not show a poorer outcome (Supplementary Fig. S18), which supports the hypothesis that 3p gain drives poor prognosis specifically through inducing cisplatin resistance.

## Discussion

We show that patterns of large low-amplitude chromosomal gain and loss are remarkably similar throughout solid cancer types, and that the frequency of whole-arm gains across cancer types is correlated with the frequency of amplifications originating from the same chromosomal arms. This structural and data-driven approach suggests that recurrent chromosomal gains provide tumors with a selective advantage, which is frequently related to established oncogenes on these chromosomes, implying that large low-amplitude gains can be of significant clinical relevance. In line with this, cell lines with low-amplitude gains of oncogenes show increased dependency on these genes and, concordantly, show increased sensitivity to inhibitors targeting these genes. Finally, we show several novel low-amplitude copy number changes that are associated with drug sensitivity and patient outcome, which underlines the notion that systematic analysis of the tumor copy number landscape can help identify valuable new biomarkers for prognosis and treatment of cancer.

Several seminal studies have previously described the pan-cancer landscape of CNAs, where they identify preferential gain or loss of specific chromosomal arms and correlations between arm level and focal copy number events [2,44]. Our analysis with large pan-solid cancer datasets builds upon this and confirms that these observations are highly reproducible and similar between datasets and that the same concepts are true within most epithelial cancer types. Moreover, our analysis indicates that oncogenes are important drivers of recurrent low-amplitude chromosomal gains. This fits well with recent findings that show increased cellular fitness as the most important driver of general aneuploidy [6] and specific chromosomal gains [8].

One contestable point is our use of specific thresholds to distinguish between low-amplitude and high-amplitude gains (amplifications). This distinction is used in the well-established GISTIC algorithm [3]; however, we know that the spectrum of copy number levels is more continuous than this binary classification suggests [45]. Notwithstanding the specific thresholds, it is evident that low-amplitude copy number gains are often overlooked when CNAs are considered as biomarkers because gains below alike thresholds are often excluded from systematic analysis, exemplified by only reporting gene-specific high-amplitude gains for large genomic studies in cBioPortal [9]. Moreover, the thresholds that we use, which are similar to the ones from GISTIC, are conservative compared to thresholds that are used in clinical practice [46, 47].

We present a list of several candidate genes that could drive the oncogenic advantage of various chromosomal gains (Supplementary Table S2). The limited amount of genes identified and the enrichment of known oncogenes strongly suggest that established oncogenes play a key role in the selective advantage of recurrent low-amplitude chromosomal gains. The list is conceivably not comprehensive. For example, the oncogene *MDM4* is not detected in our analysis, while it has been shown to be an important driver of chromosome 1q gain through inactivation of P53 [6,48]. It is possible that this relationship is not detected due to other confounders, such as *TP53* mutations, which are very frequent in the cell line panel used. The observation that 1q gain also frequently occurs in *TP53* mutated tumors also suggests that there are more 1q genes that provide a selective advantage when gained.

Besides the annotated oncogenes, we also list several genes with a less well-established role in cancer. *TGIF1* is the most frequently gained gene on chromosome 18p and shows a pattern with significantly increased dependency on this gene when gained. Its expression has been shown to have a growth-promoting effect in various cancer types [49–51], while *TGIF1* is not present in OncoKB or the Cancer Gene Census. Our results suggest that increased *TGIF1* expression through low-amplitude copy number gain could have an oncogenic effect. The same might hold true for components of the cytoskeleton, since *TUBB*, *ACTB*, and *ACTG1* are all included in the candidate gene list, which would be in line with the frequently observed increased expression of cytoskeletal proteins in cancer and associations with poor outcome and chemoresistance [52, 53]. Finally, the five genes that were identified in all gene dependency datasets but are not annotated as oncogenes (*ACSL3*, *PPP1CA*, *PP1R15B*, *RAB6A*, and *TEAD1*) represent promising candidates for an oncogenic role through low-amplitude copy number gain.

We show that cancer cell lines with increased copy number of oncogenes show increased dependence on these genes. For some oncogenes like *BRAF* and *KRAS*, this effect is relatively minor compared to the effect of activating mutations. Actually, one of the key roles of low-amplitude gain might be to increase the number of mutant oncogene copies, as for *BRAF*, where we observed a significant difference in dependency based on copy number status only in cell lines with hotspot mutations. Nevertheless, there are other genes where activating mutations are infrequent and where the effect of copy number is much more pronounced, such as *MYC* and *E2F3*. Similarly, although the difference is small and may not be therapeutically relevant, cell lines with gain show increased sensitivity to targeted compounds like EGFR and BRAF inhibitors. For other inhibitors, like BCL2 inhibitors, we describe that gain of these regions has a more pronounced effect and could possibly be used as a biomarker to improve upon existing stratification methods for these compounds.

We identify 32 low-amplitude gains that are identified in two independent datasets as possible biomarkers for drug sensitivity (13/32) or resistance (19/32). This suggests that CNAs might be applicable as biomarkers more broadly than mutations and amplifications, which are almost always associated with sensitivity (51/53 in our analysis). Of the identified gains, seven overlap with RACs in the GDSC2 analysis, and only two of them are also identified as significant there, while the overlap for mutations was much higher (21/22) [15]. The limited overlap could be due to the exclusion of hematological malignancies in our analysis, although this does not explain why the mutations do show good concordance. One of the strongest associations was between gain of 6p25 (3–4 Mb) and sensitivity to IAP inhibition, which could be used to improve upon existing stratification for these compounds [32]. The established role of the *RIPK1* and *TNF* genes, located on chromosome 6p, in the IAP pathway [54] suggests that increased expression of these genes through low-amplitude copy number gain could drive the sensitivity of cell lines with extra copies to IAP inhibition.

In multiple datasets, we identified associations between (i) gain of chromosome 1q in neuroblastoma, poor survival, *MCL1* dependency, and *MCL1* inhibitor sensitivity and (ii) gain of chromosome 3p25 and cisplatin resistance in cell lines and poor prognosis in malignant germ cell tumors and lung cancers. *MCL1* has been described before as the driver of 1q gain in other cancers [2], but was not linked to treatment with MCL1 inhibitors. Despite the established relevance of MCL1 in neuroblastoma biology and therapy response, and 1q gain as a marker of poor prognosis [55–57] this gain was not previously linked to *MCL1* activity in neuroblastoma. The association between gain of 3p and cisplatin resistance has been described before in nonseminomatous malignant germ cell tumors [40], and here we show that it is also associated with poor outcome in lung adenocarcinoma. The 3p25 region is lost in the majority of lung cancers [58], but increased copy numbers of the *VHL* and *FANCD2* genes in the 3p25 region have recently been reported to be associated with high-grade lung adenocarcinomas that seem to follow a different genetic evolutionary trajectory that does not involve 3p loss [59].

Overall, our data strengthen the hypothesis that the general aneuploidy landscape, specifically for chromosomal gains, is shaped by oncogenic advantages of individual aberrations and suggests that a large part of the genes involved are established oncogenes. The other genes in our candidate list provide a valuable starting point for the identification of novel oncogenes that exert their effect through low-amplitude copy number gains. Finally, the specific examples that we describe show that systematic analyses of low-amplitude CNAs have the potential to identify clinically relevant biomarkers in cancer.

## Supplementary Material

gkaf689_Supplemental_Files

## Data Availability

Copy number segments, clinical data, and GISTIC calls (CNA genes) from the TCGA pan-cancer series and MSKCC Impact were downloaded from cBioPortal [9]. Data from the CCLE were downloaded from the DepMap data portal. A list of all data used is shown in Supplementary Table S1. Source code for all analyses can be found at Figshare DOI: https://doi.org/10.6084/m9.figshare.28956854.
