# Supplementary material for: Low-amplitude copy number gains shape cancer through known and novel oncogenes with associated therapeutic vulnerabilities
Source: Nucleic Acids Res. 2025 Jul 22;53(14):gkaf689. doi: 10.1093/nar/gkaf689 (PMC12282949; doi:10.1093/nar/gkaf689)
Supplement: gkaf689_Supplemental_Files [file gkaf689_supplemental_files.zip › NAR-03379-N-2024.R1_supplementary_figures.pdf]

Supplementary Figure 1

A

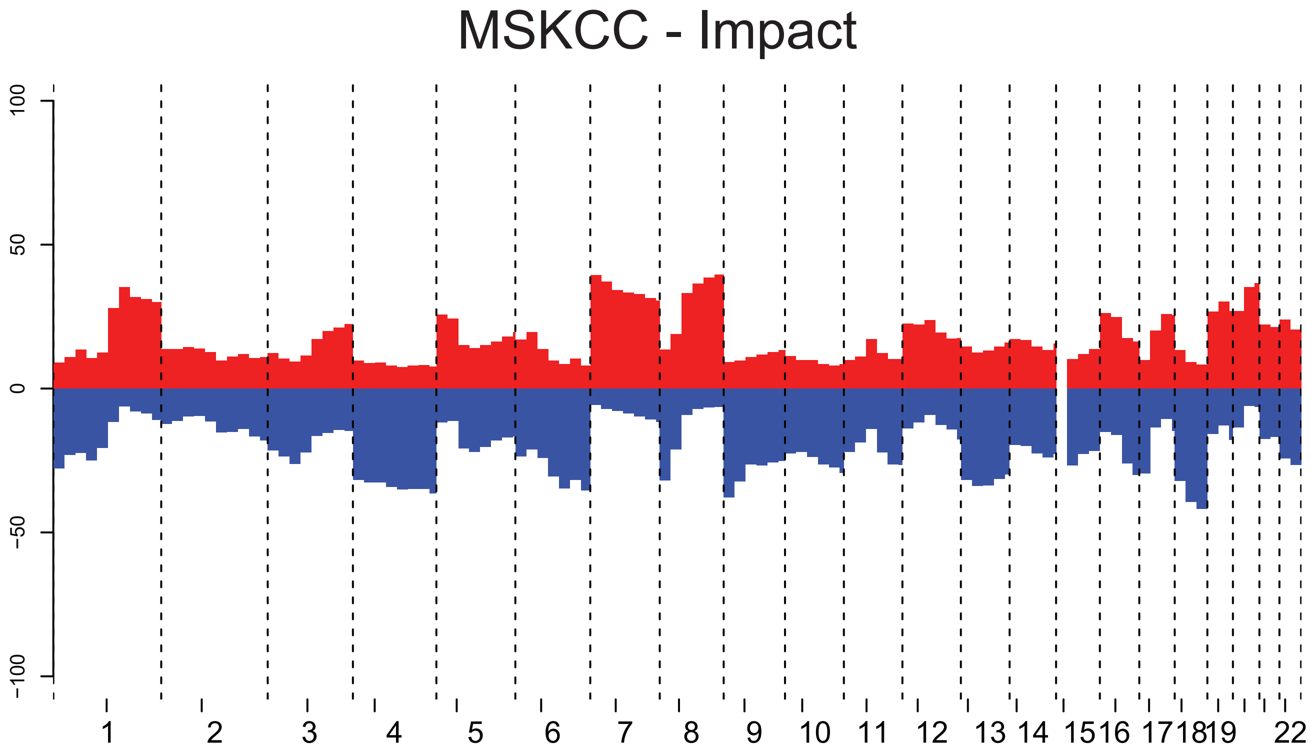

B

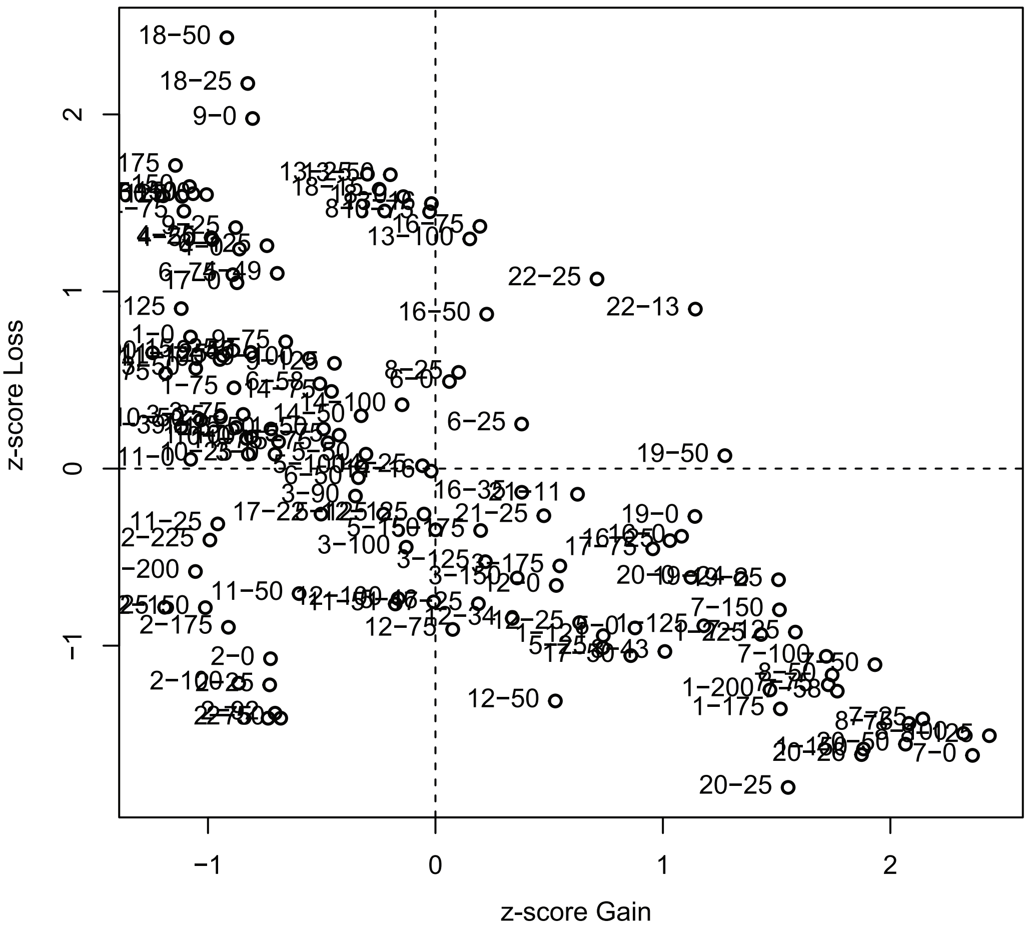

Supplementary Figure 1: Related to Figure 1A,B. Broad CNA's in the MSKCC-Impact dataset. A) Frequency plot of broad gains (red) and losses (blue). B) XY-plot showing the z-scores for loss and gain for each genomic 25Mb bin, indicated by the chromosome number and starting location in Mb.

Supplementary Figure 2

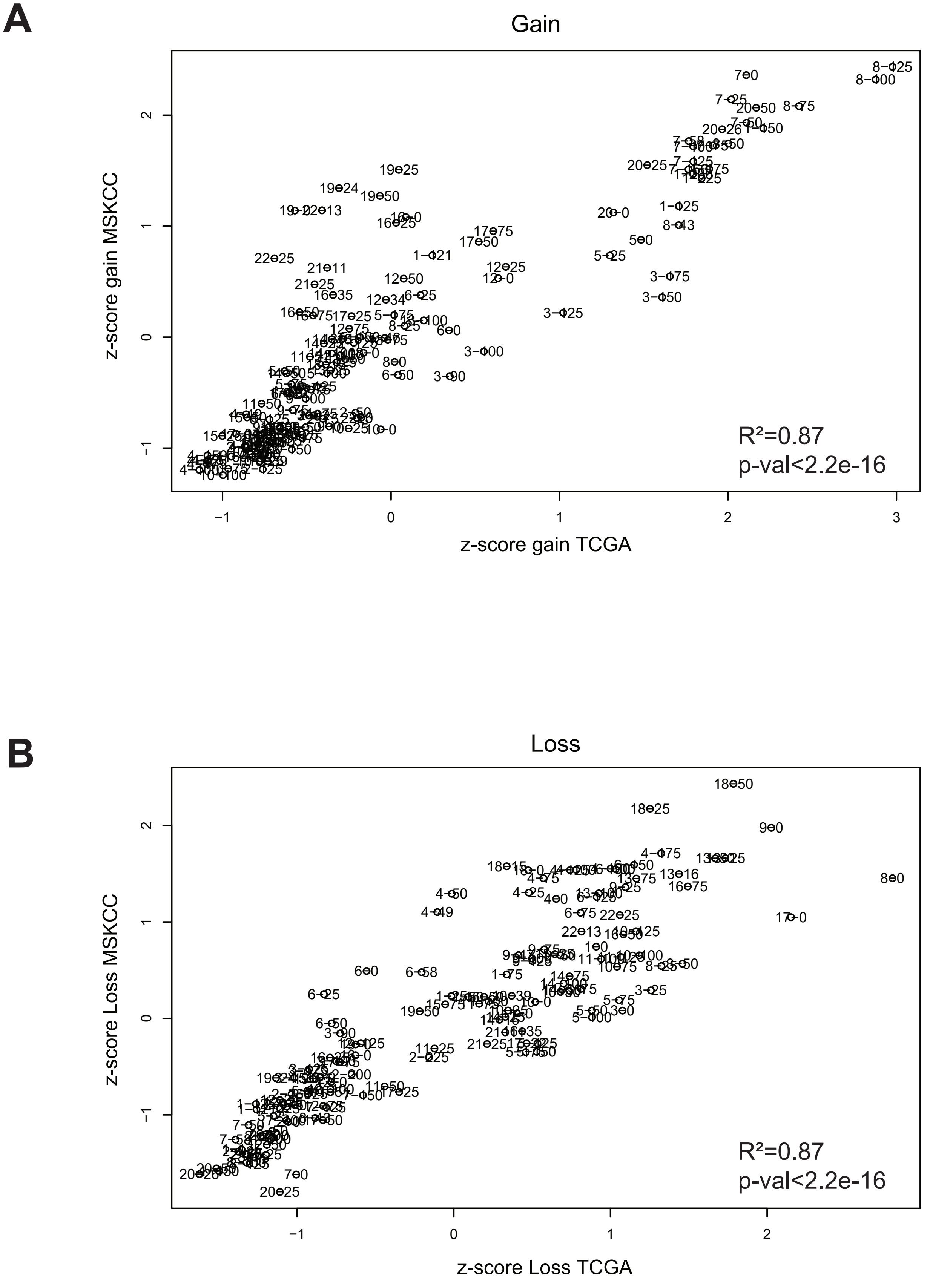

Supplementary Figure 2: Comparison of broad CNA's between the TCGA and the MSKCC-Impact series. A) XY plot of z-scores for gain for each genomic 25Mb bin, indicated by the chromosome number and starting location in Mb in both series. B) XY-plot showing the z-scores for loss for each genomic 25Mb bin.

Supplementary Figure 3

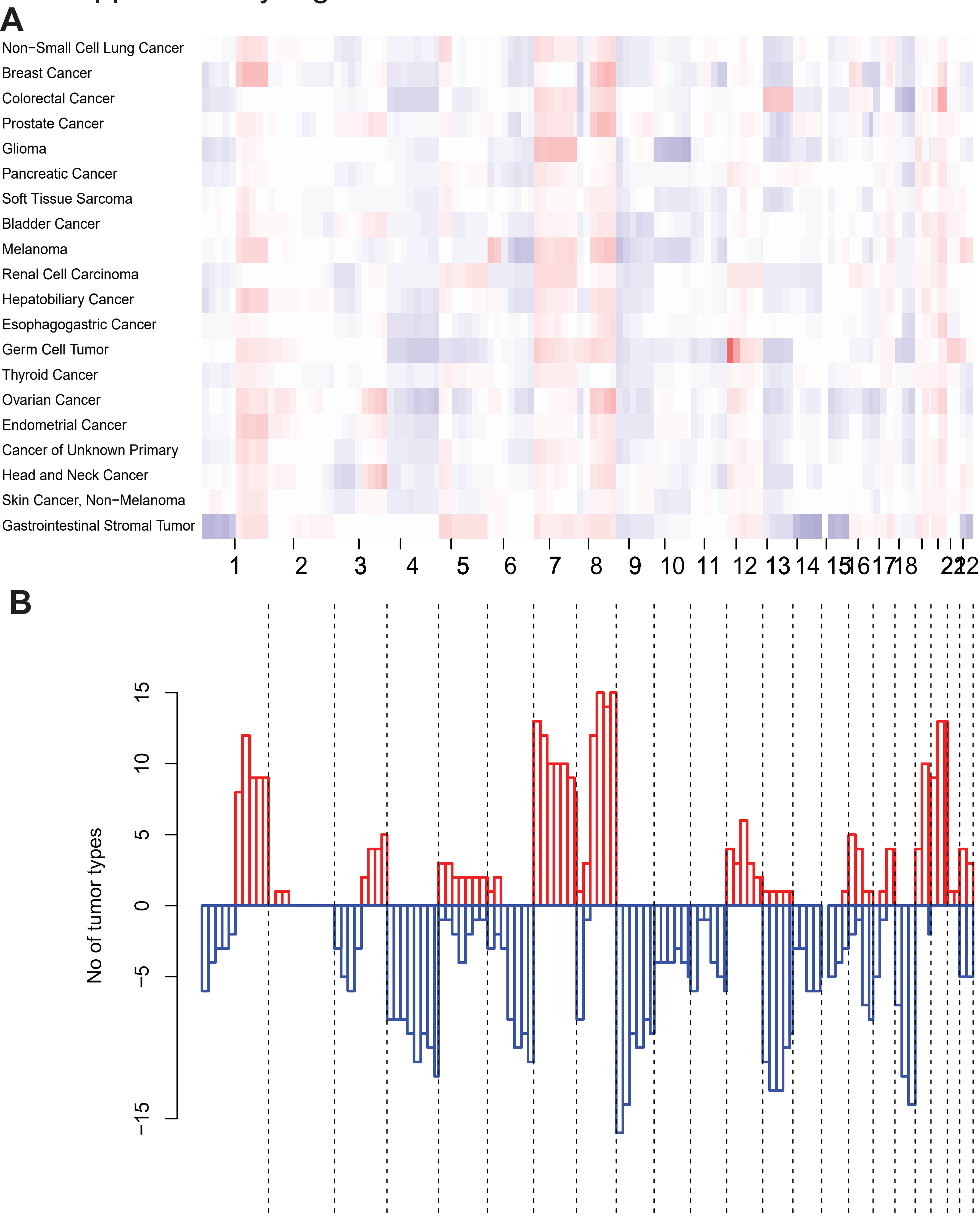

Supplementary Figure 3: Related to Figure 1C. Tendency for gain (red) and loss (blue) within specific tumor types in the MSKCC. A) Colored bars represent the mean copy number per bin in the specified tumor type. B) Bars represent the number of tumor types that show a frequency of >30% of gain (red) or loss (blue).

Supplementary Figure 4

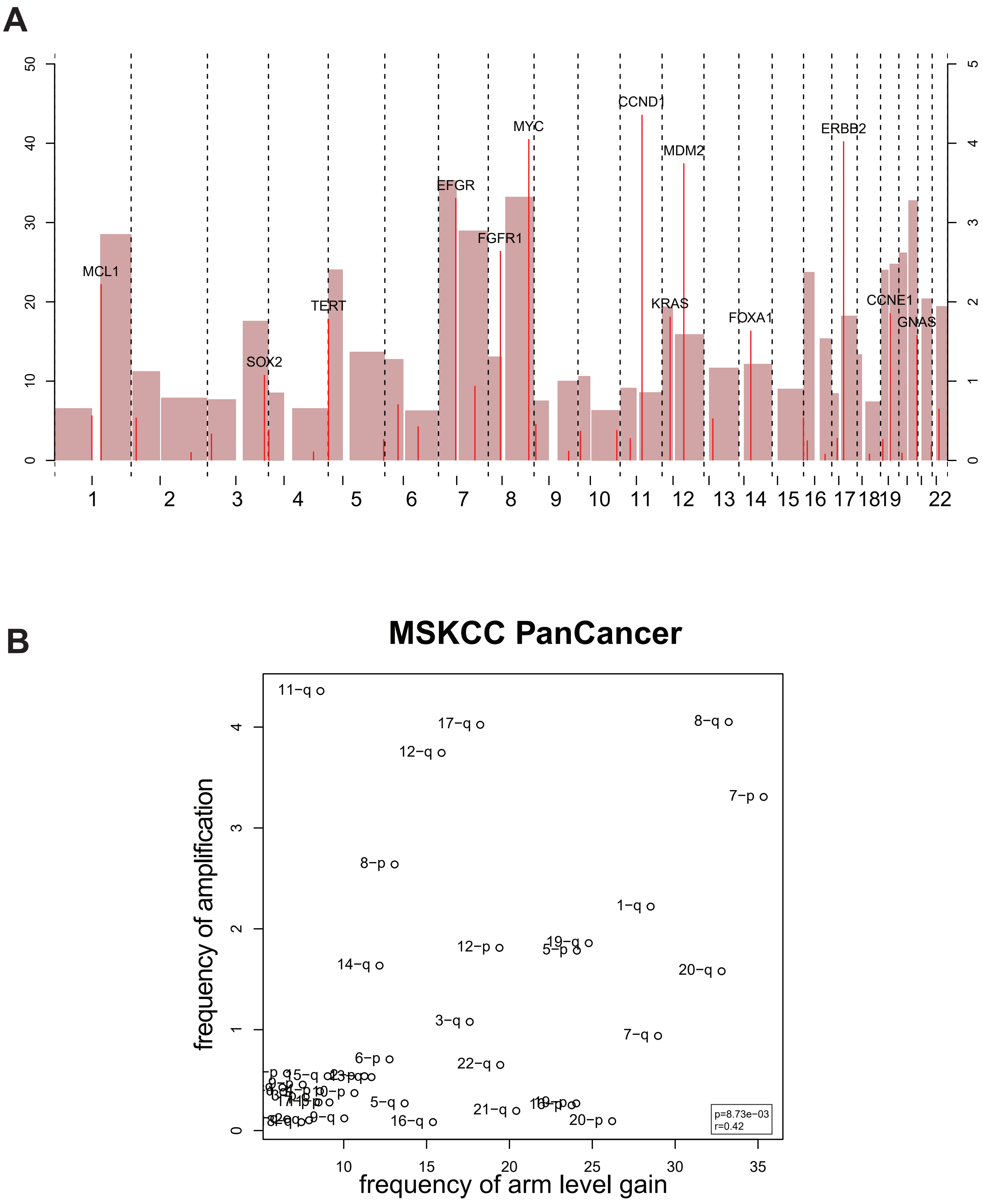

Supplementary Figure 4: Related to Figure 1D,E. A) Frequency plot of arm level gains (dark red, left y-axis) and the most frequent focal gains per chromosome arm (bright red, red y-axis). B) XY-plot showing the correlation between the arm level and focal gains per chromosome arm shown in A.

Supplementary Figure 5

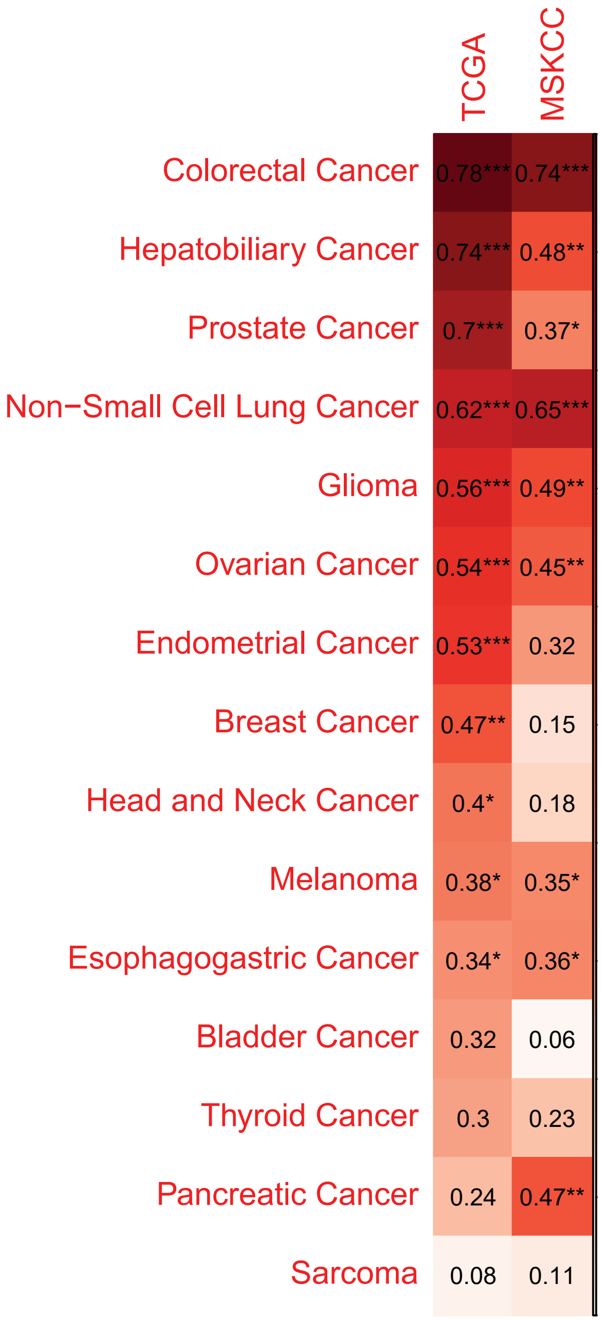

Supplementary Figure 5: Correlations of arm level gains and focal gains in tumor types that overlap between the TCGA and MSKCC-Impact. Higher color intensity reflects higher r-values. Significance is denoted by asterisks (* p<0.05, ** p<0.01, *** p<0.001).

Supplementary Figure 6

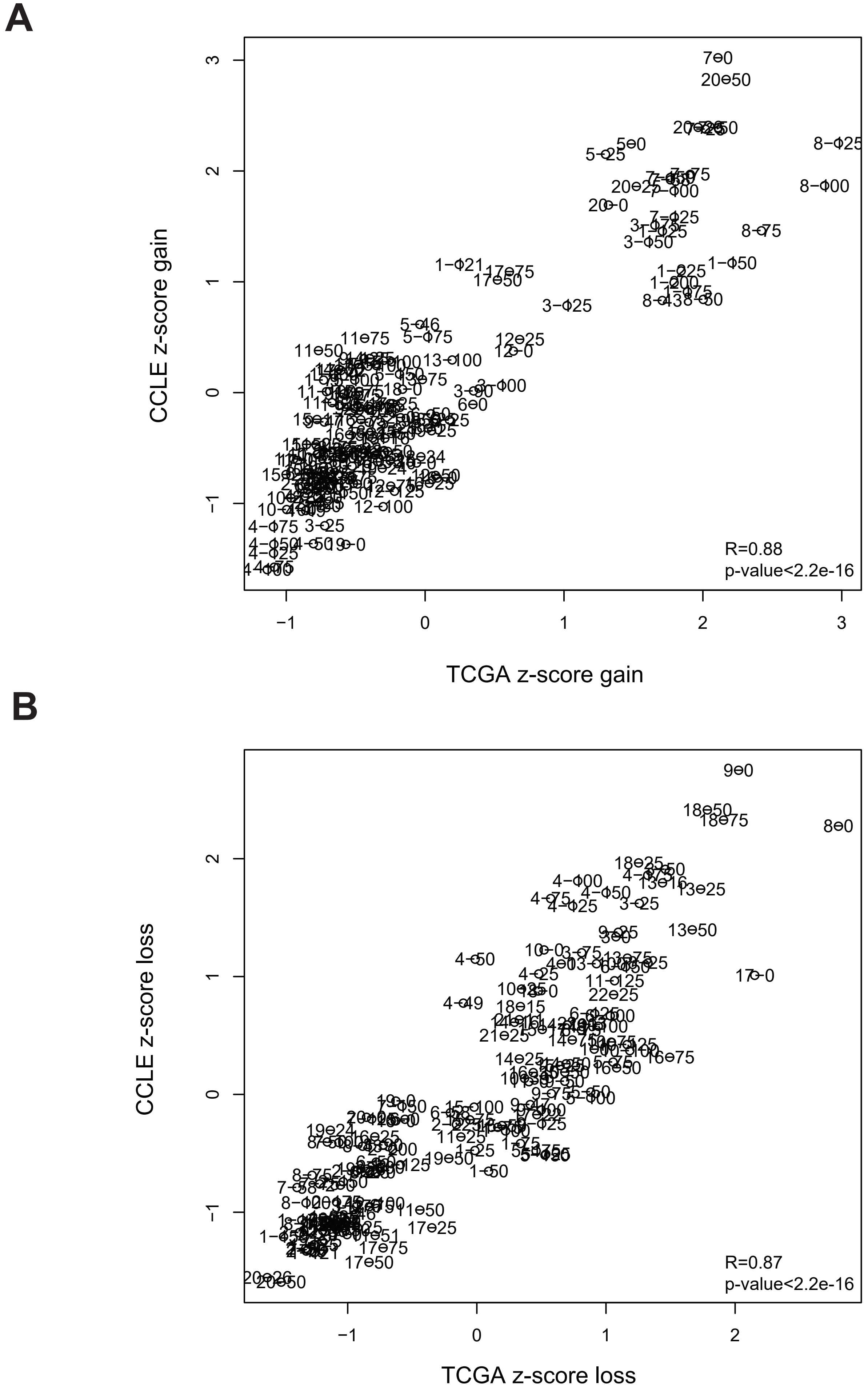

Supplementary Figure 6: Comparison of broad CNA's between the TCGA and the CCLE series. A) XY plot of z-scores for gain for each genomic 25Mb bin, indicated by the chromosome number and starting location in Mb in both series. B) XY-plot showing the z-scores for loss for each genomic 25Mb bin.

Supplementary Figure 7

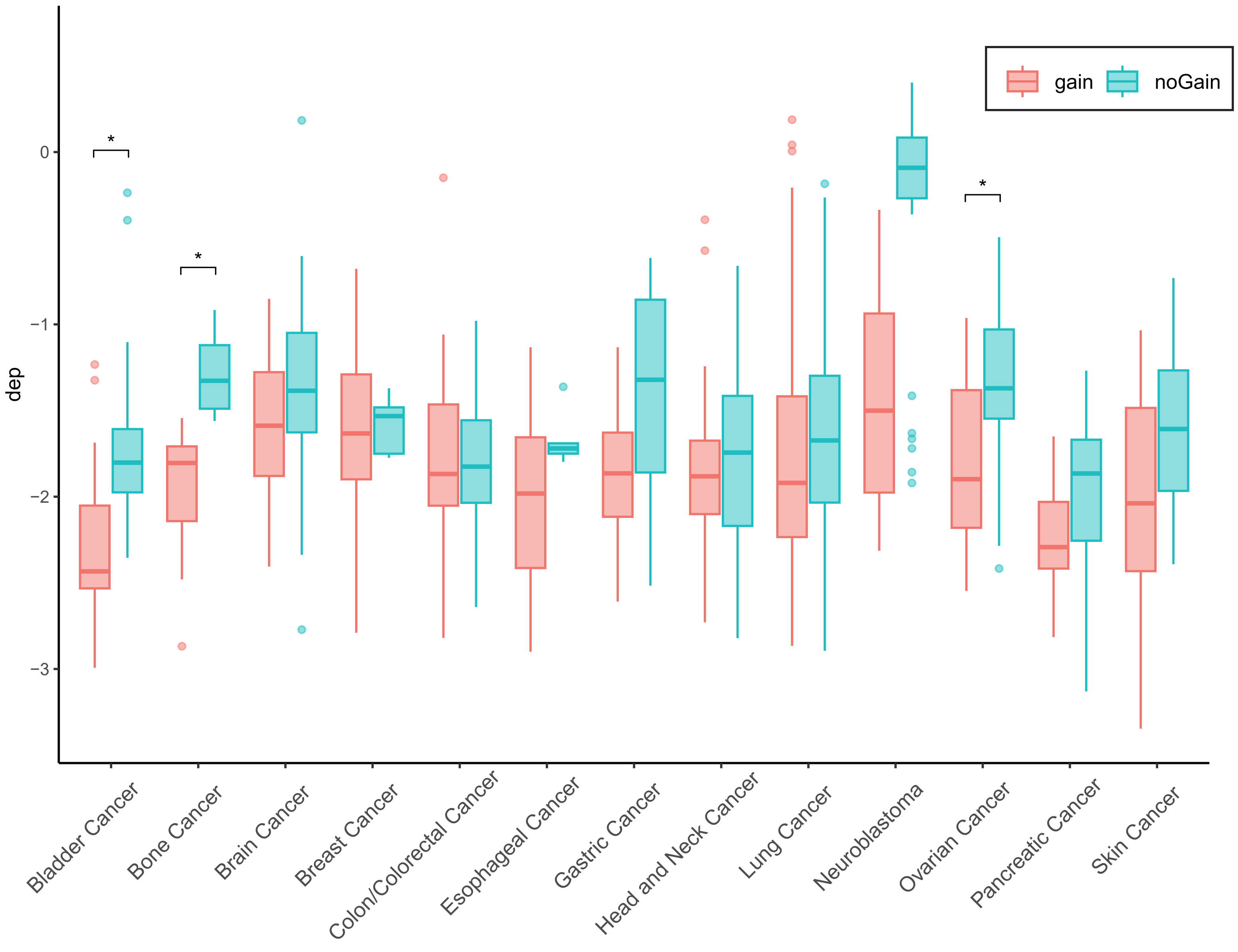

Supplementary Figure 7: Boxplot of MYC dependency in cell lines of specific tumor types with or without gain of the MYC region. Cell lines with amplification were not taken into consideration. Asterisks indicate significant differences (t-test, multiple testing correction fdr).

Supplementary Figure 8

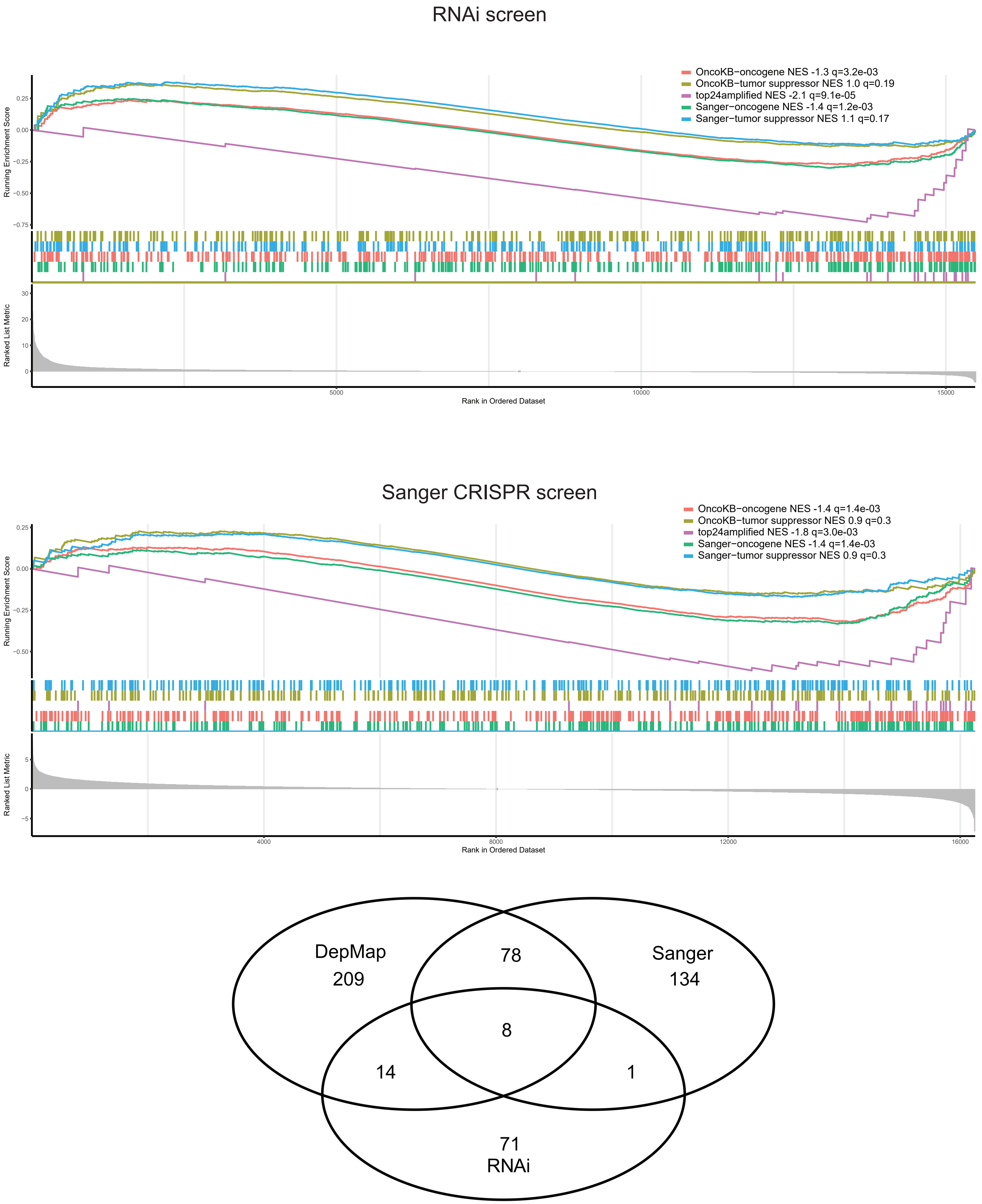

Supplementary Figure 8: Related to Figure 2. GSEA of genes that show increased dependency when gained in the A) RNAi and B) Sanger CRISPR screening dataset. Genes are ordered based on the log10(q-value), with the sign determined by the dependency difference. Curves represent running enrichment scores of the indicated genes sets in the ranked data. Colored bars represent the position of the genes in the respective sets in the ranked list. C) Venn diagram showing overlap between the included genes in the used datasets.

Supplementary Figure 9

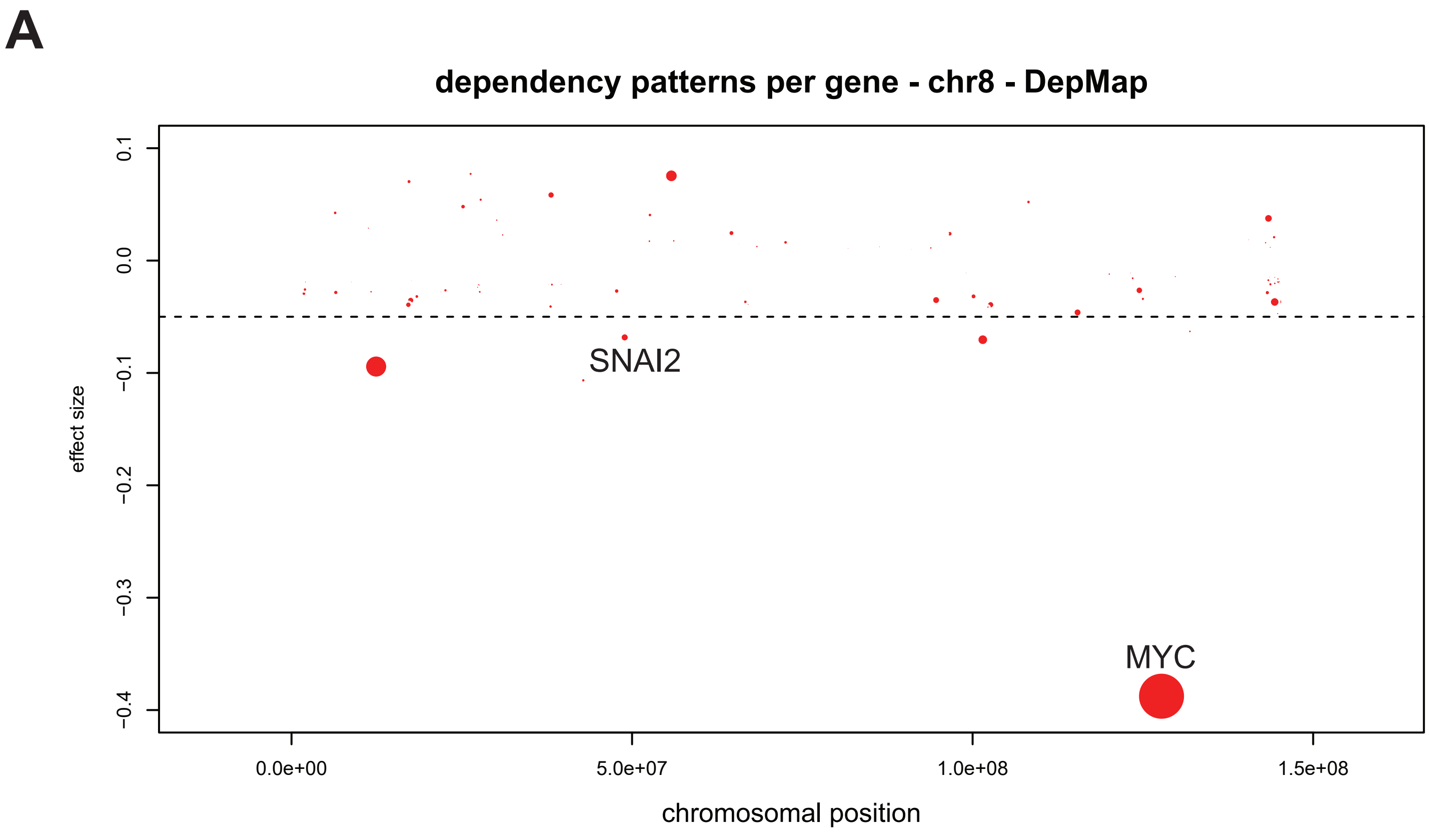

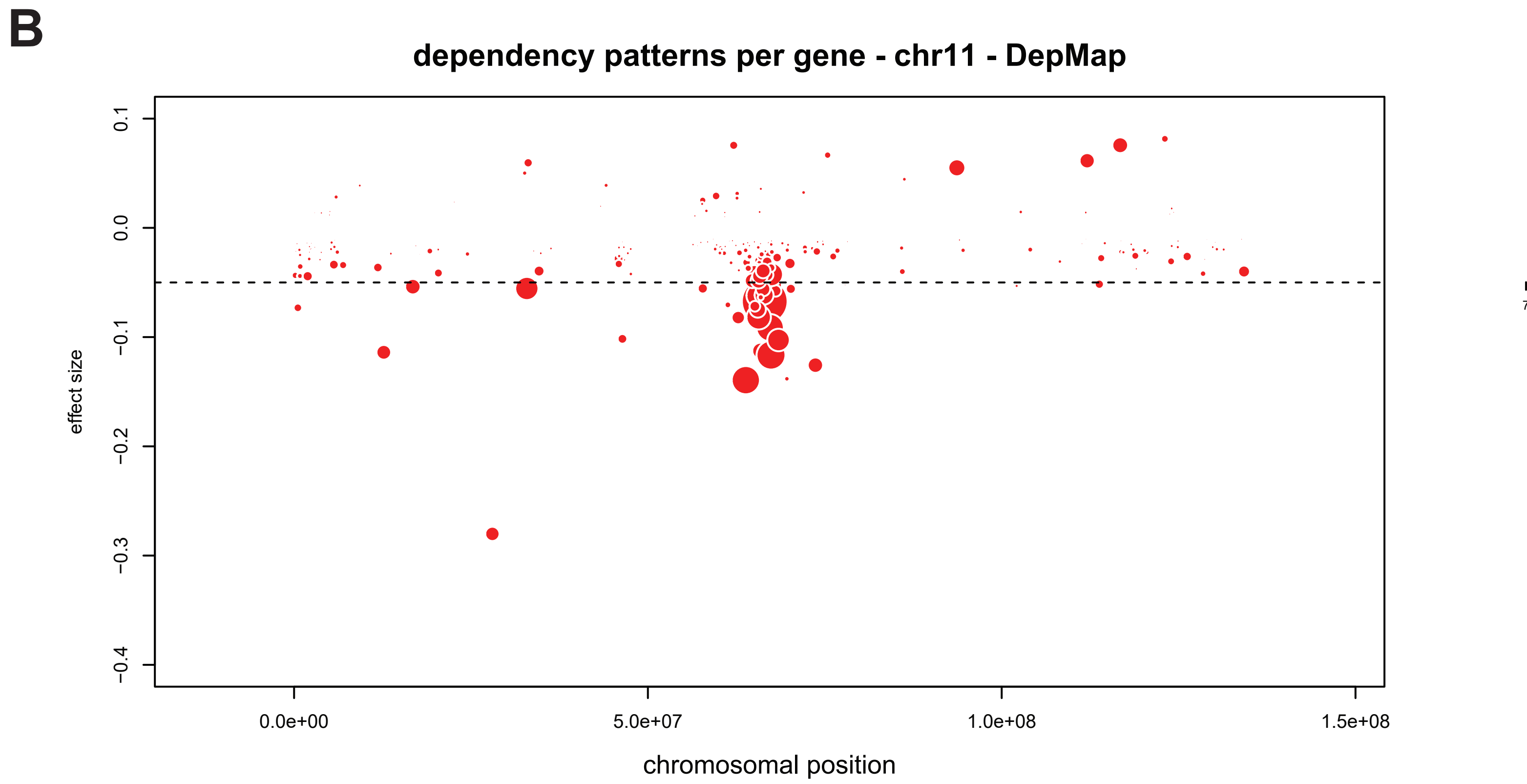

Supplementary Figure 9: Dotplots showing the difference in dependency for all genes in cell lines with gain versus cell lines with a neutral copy number on chromosome 8 (A) and 11 (B). The size of the dots represent the -log10 of the FDR q-value.

Supplementary Figure 10

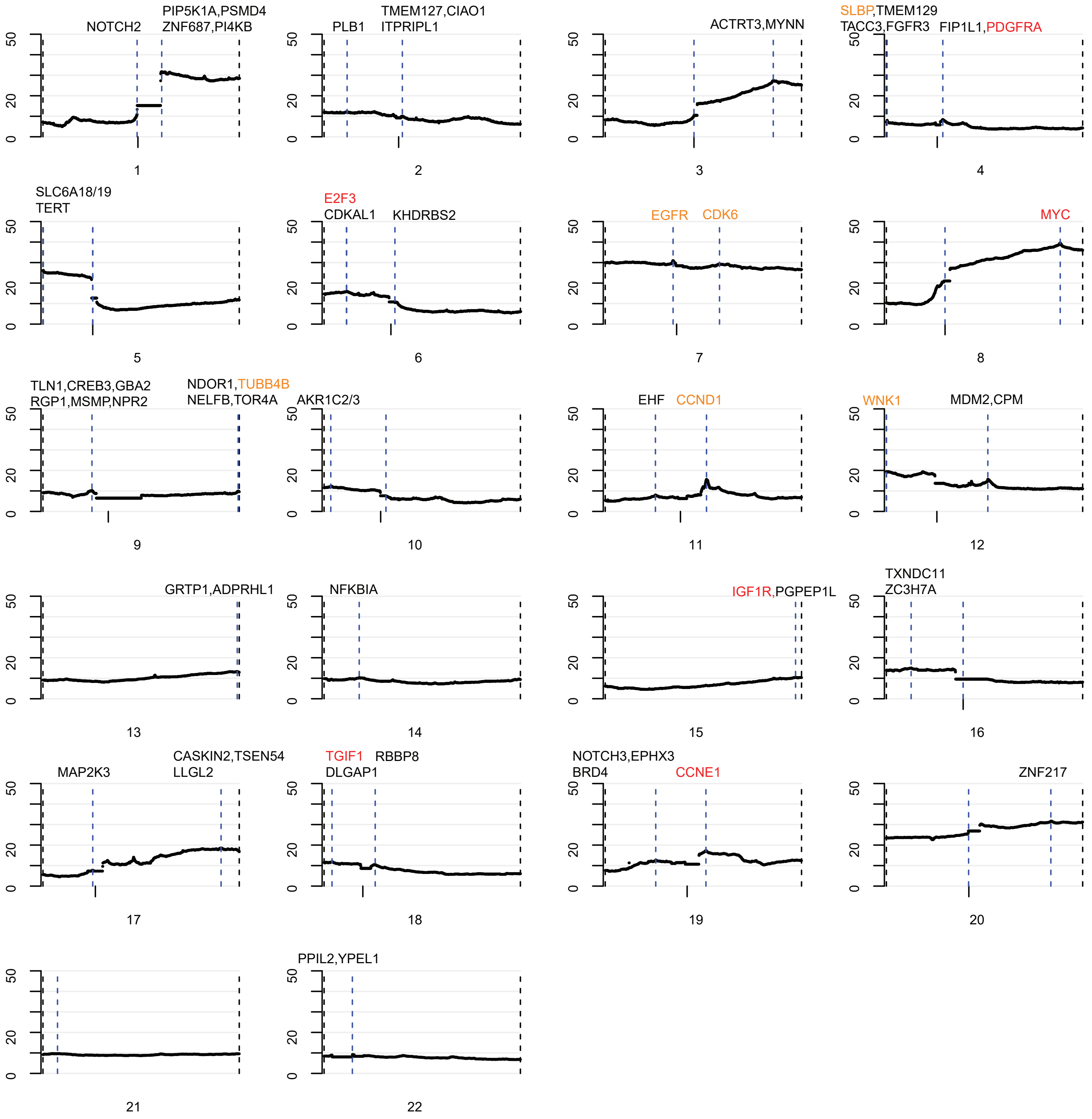

Supplementary Figure 10: For each 100kb bin the frequency of gain was calculated in the solid tumors from the pancancer TCGA series. The most frequently gained bins per chromosome arm are indicated by blue dotted lines and the genes located in this region are shown above. Genes also identified as candidates in Supplementary Table 1 (identified as showin differential dependency in 2 or more datasets) are colored in red and genes identified in 1 dataset are shown in orange.

Supplementary Figure 11

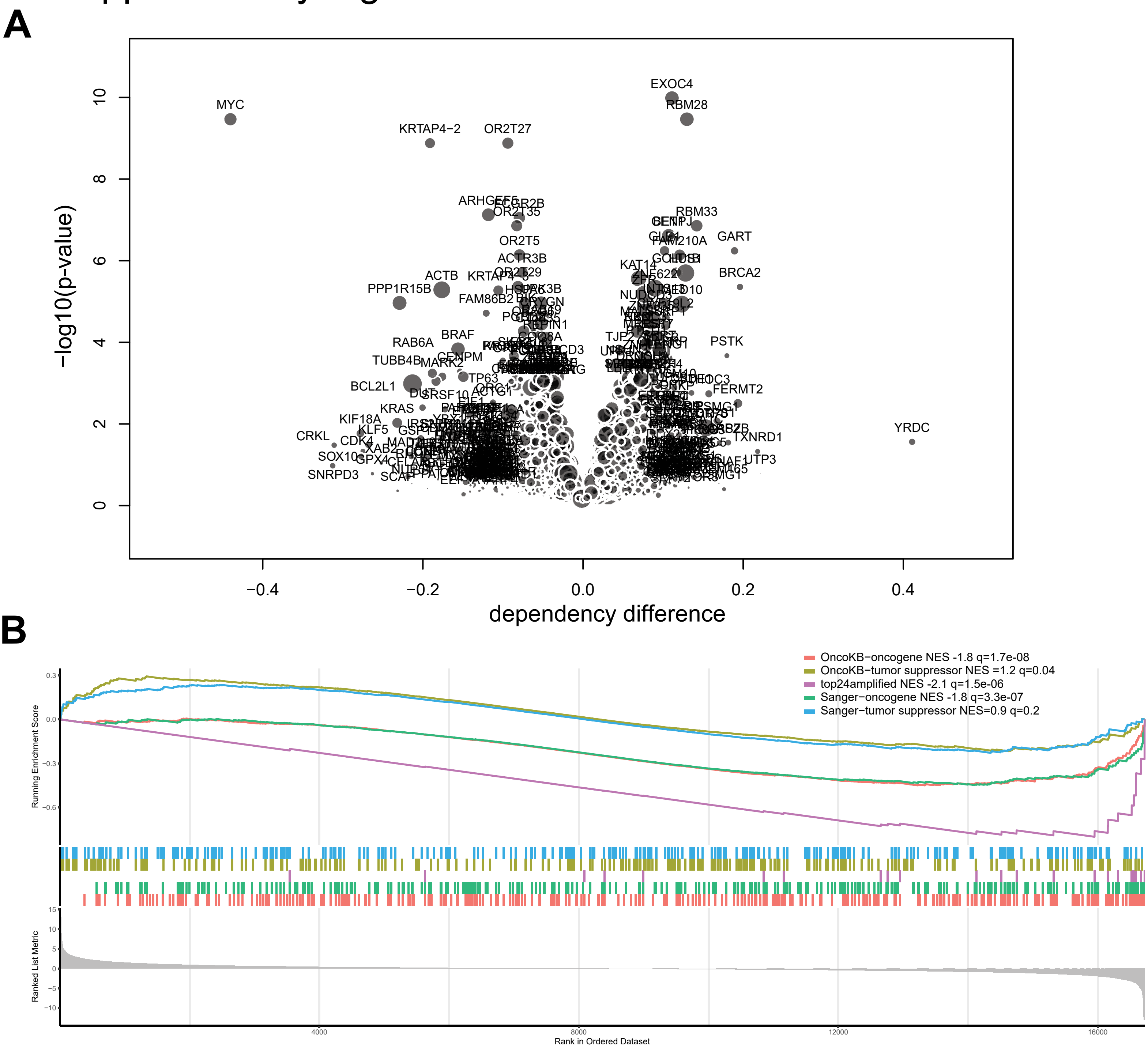

Supplementary Figure 11: Related to Figure 2. A) Volcano plot showing differential CRISPR dependency based on arm level gain of the gene. Dot size is relative to the frequency of gain of this region. Genes on the left show higher dependency in cell lines with copy number gain. B) GSEA of the analysis depicted in A.

Supplementary Figure 12

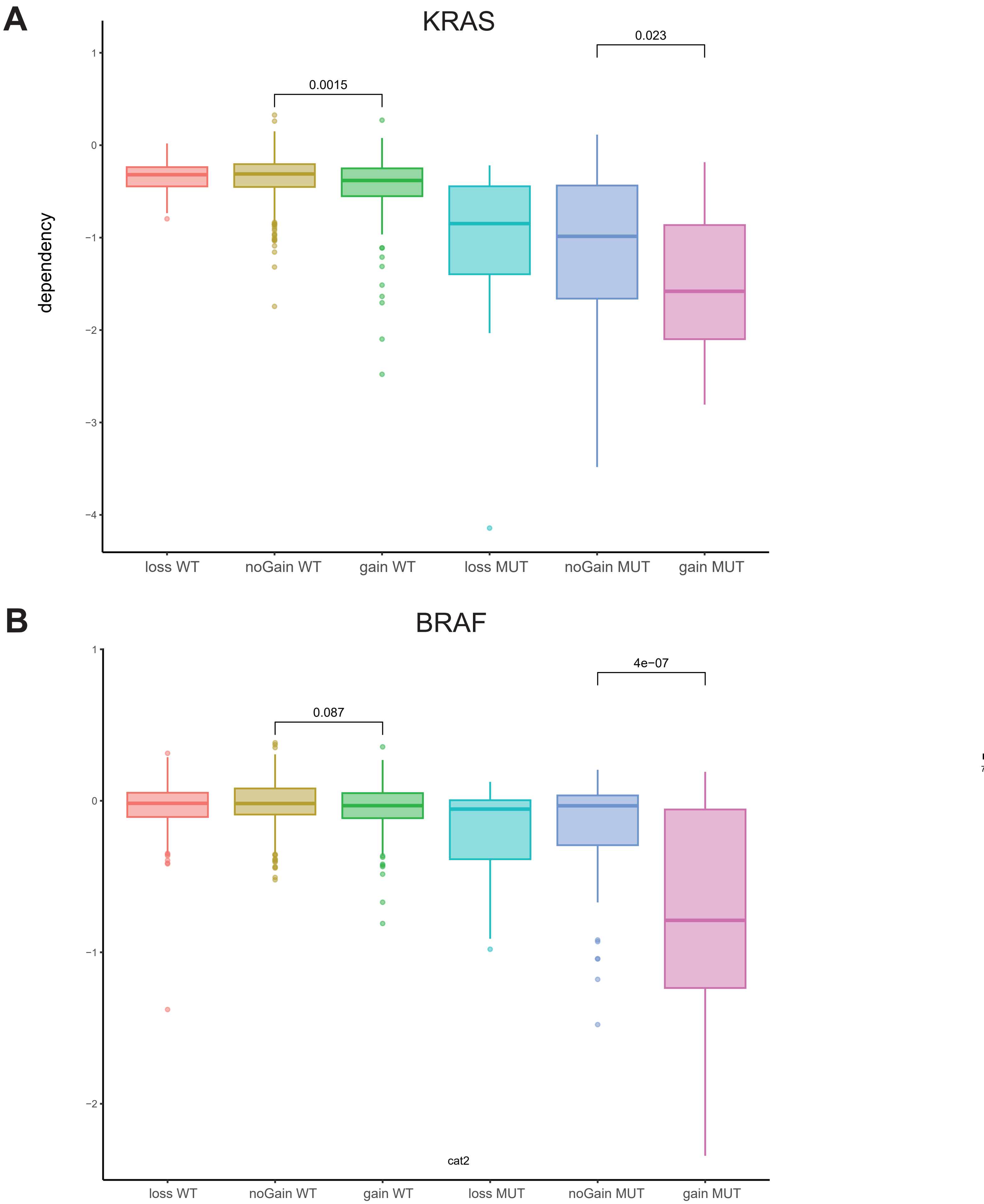

Supplementary Figure 12: Boxplots showing dependency of cell lines based on combined hotspot mutation and low level copy number status for A) KRAS and B) BRAF in the DepMap CRISPR screen. Indicated p-values were generated by t-test.

Supplementary Figure 13

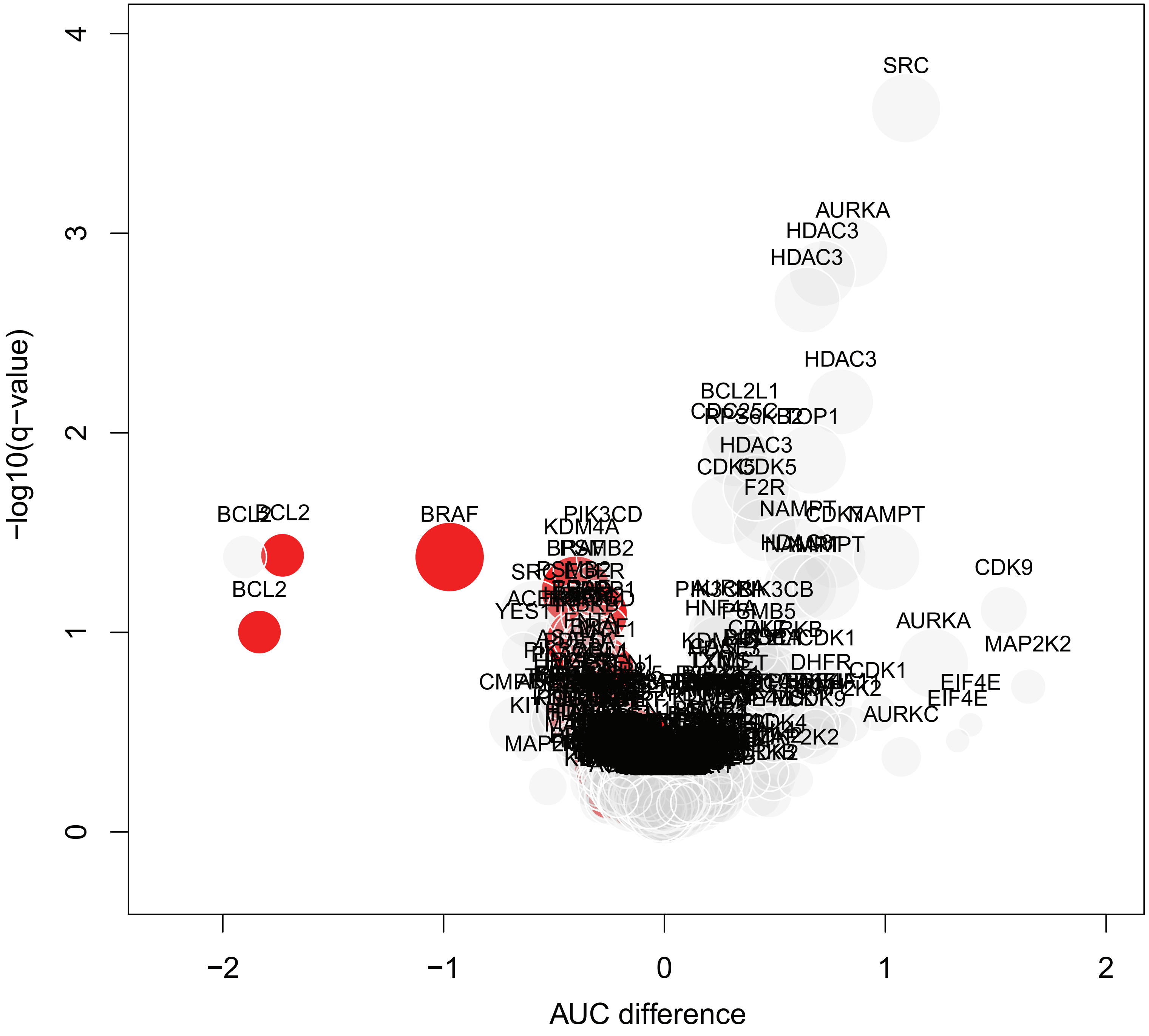

Supplementary Figure 13: Related to Figure 3. Volcano plot showing differential drug sensitivity for drugs based on the low level gain of their target gene in the CTRP2 dataset. Red colors mean drugs where AUC is strongly correlated with dependence on the target gene in the cell line panel.

Supplementary Figure 14

GDSC2

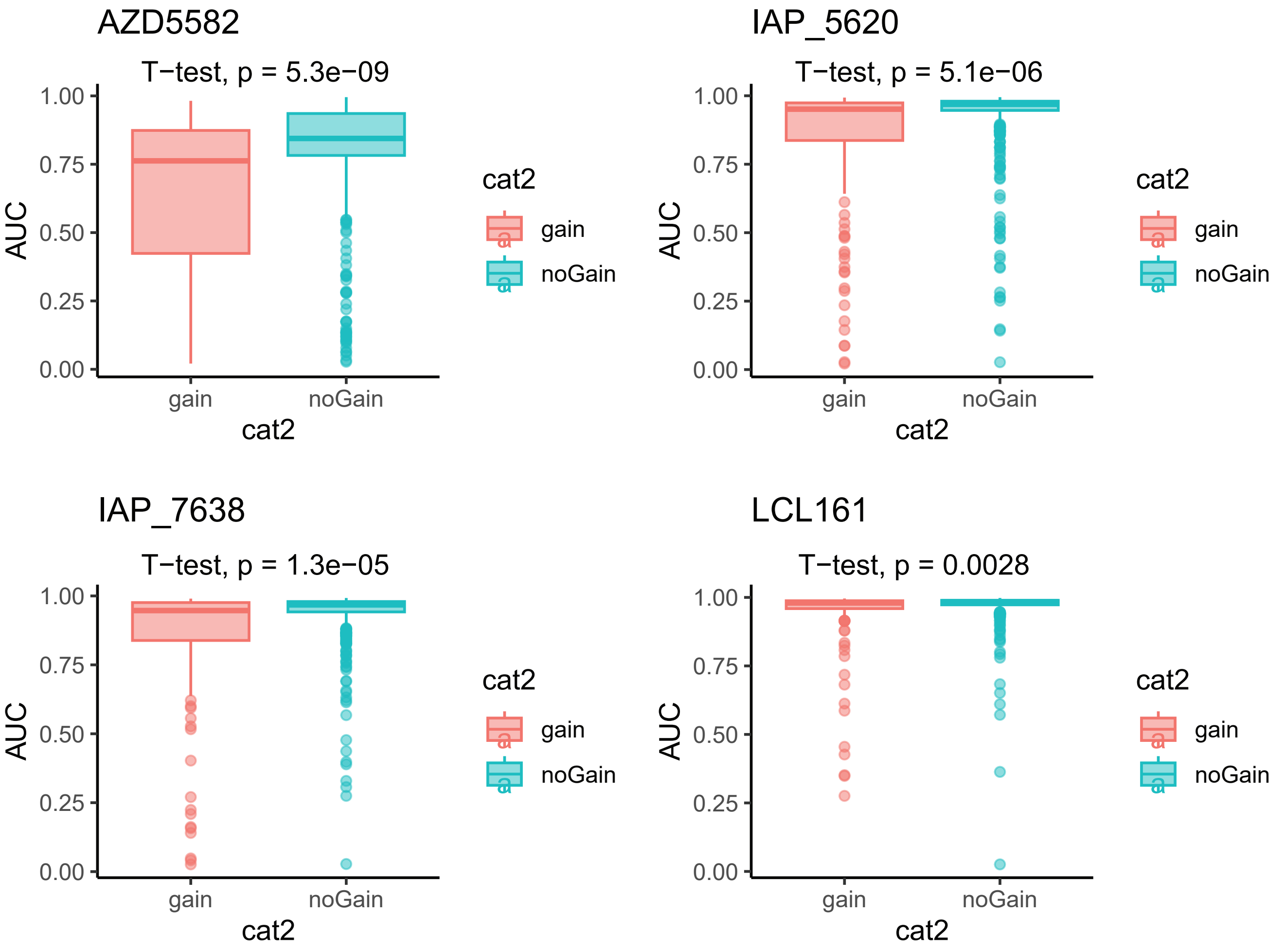

CTRP2

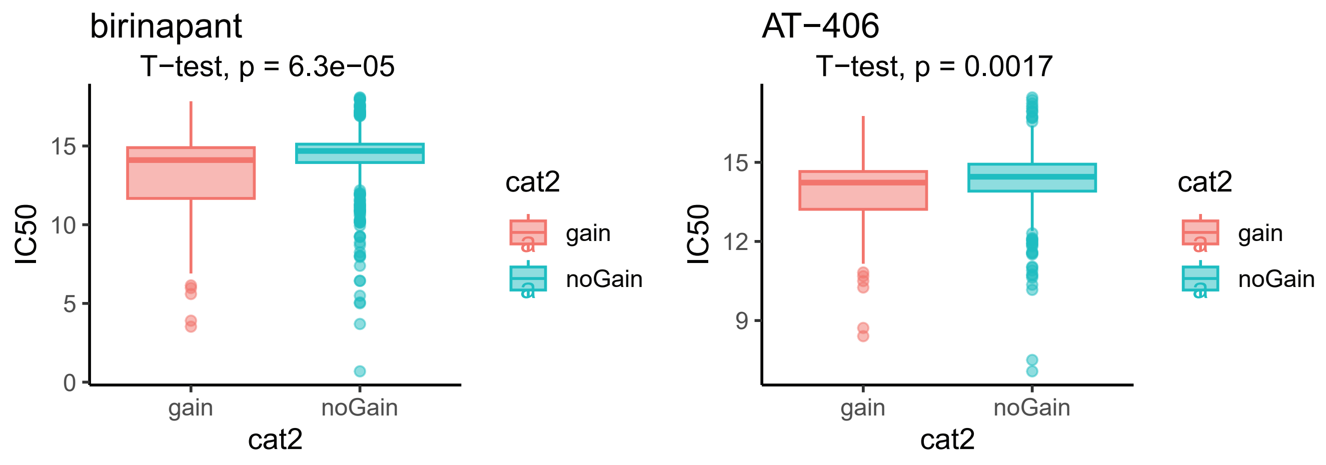

Supplementary Figure 14: Related to Figure 4. Box plots showing differential sensitivity to IAP inihbitors in the GDSC2 and CTRP2 datatsets between cell lines with low amplitude gain and cell lines without gain.

Supplementary Figure 15

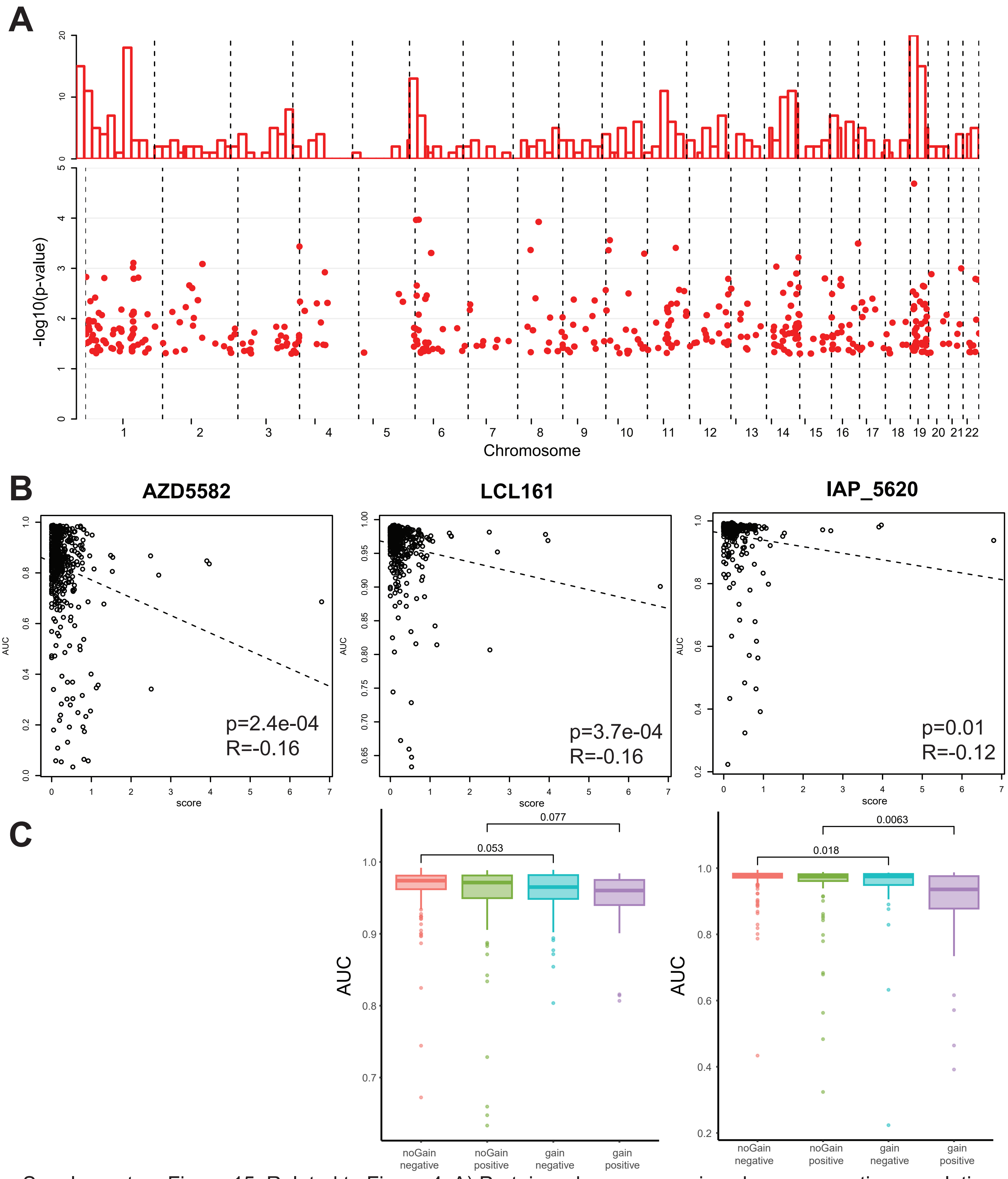

Supplementary Figure 15: Related to Figure 4. A) Proteins whose expression shows a negative correlation with sensitivity to AZD5582. Red circles represent genes and their position on the x-axis their chromosomal location. Bars above represent the number of correlated genes for every 25Mb bin. B) XY plots showing the relationship between the pairwise gene expression score suggested by Bardia et. al. and AUC of different IAP inhibitors in the CCLE. R- and p-values were generated through Spearman correlation. C) Boxplot showing AUC for the above mentioned compounds in cell lines based on their 6p gain status and gene expression score (scored as positive for all scores > the 70th percentile of observed scores). P-values calculated by Wilcoxon test are shown for relevant combinations.

Supplementary Figure 16

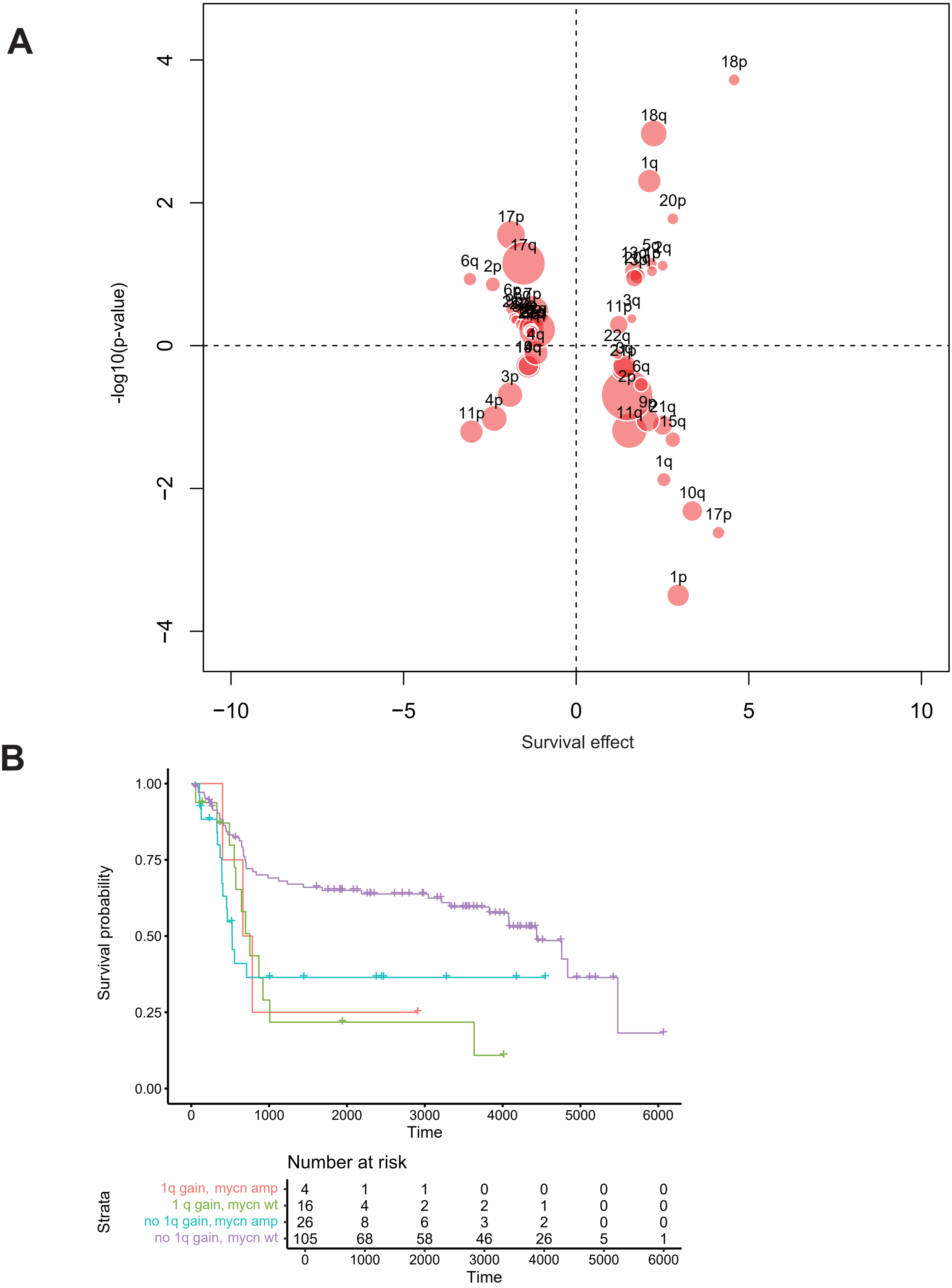

Supplementary Figure 16: Related to Figure 5D. A) Bubble plot showing the association between copy number loss (negative y-values) or gain (positive y-values) and survival of neuroblastoma patients (positive x-values signify poorer survival and negative x-values signify better survival). B) Kaplan-Meier curve showing survival of patients depending on MYCN amplification status and presence of MCL1 gain.

Supplementary Figure 17

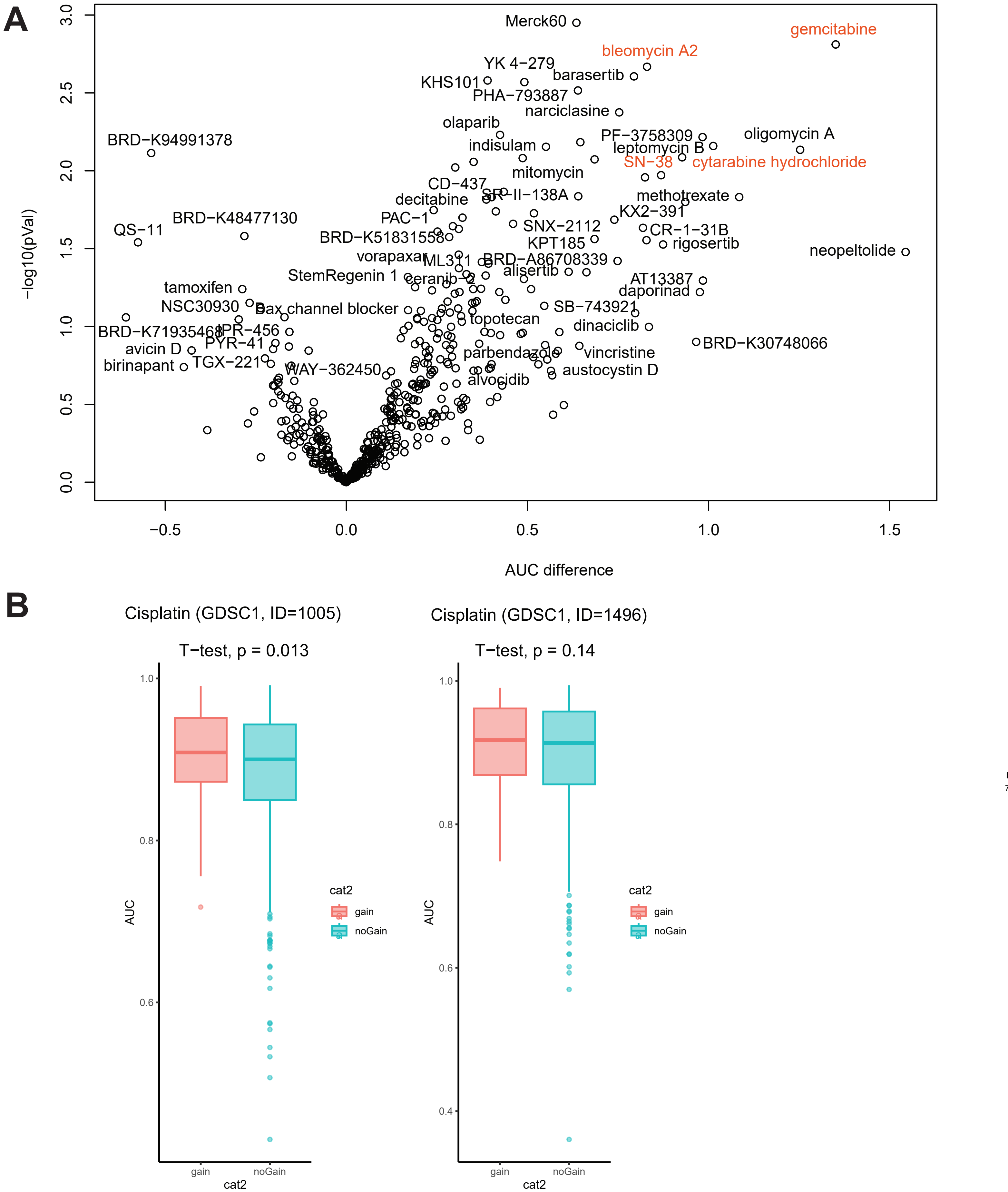

Supplementary Figure 17: Related to Figure 6B. A) XY plot showing differential sensitivity to compounds based on the 3p gain status of cell lines in the CTRP2 dataset. Every dot represent one compound with its place on the x-axis indicating resistance (x>0) or sensitivity (x<0) to this compounds in cell lines with 3p 13-14Mb gain. DNA damaging compounds are labeled in orange. B) Boxplots showing the AUC for cisplatin in cell lines with 3p 13-14Mb gain versus cell lines without in the GDSC1 dataset.

Supplementary Figure 18

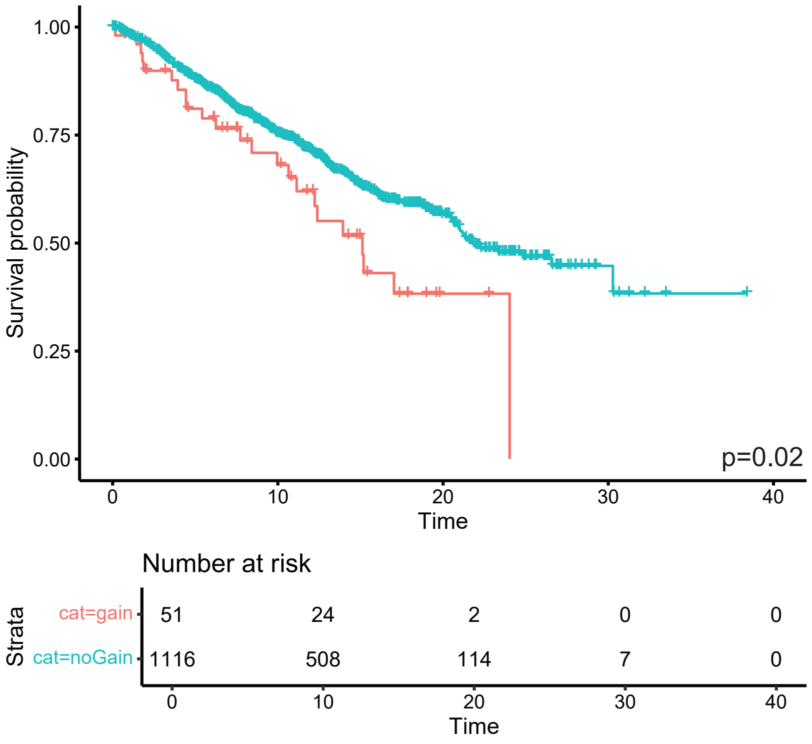

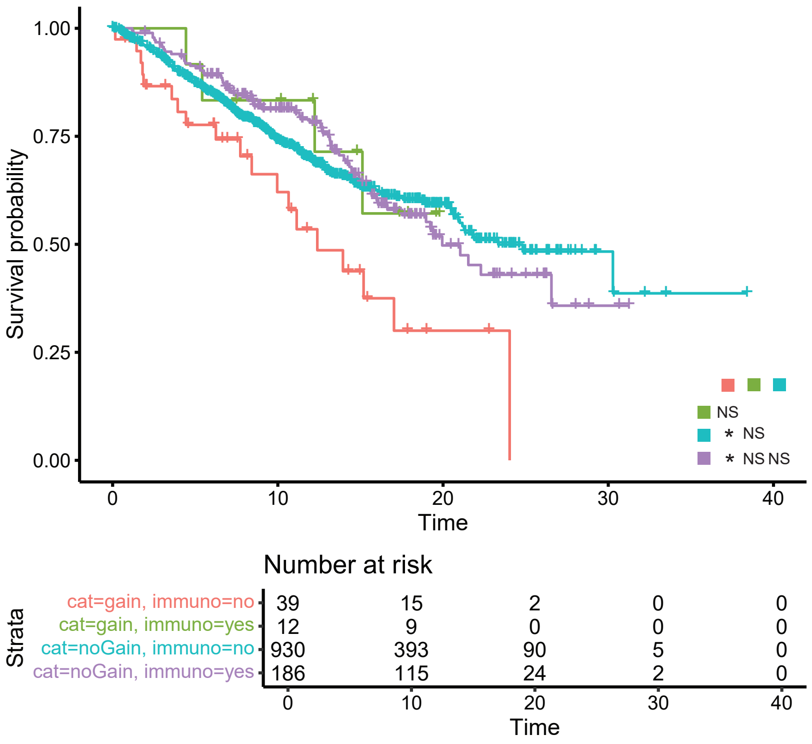

Supplementary Figure 18: Related to Figure 6E. A) Kaplan-Meier curve showing survival of lung adenocarcinoma patients in the MSKCC-Impact cohort depending on 3p gain status. B) Kaplan Meier on the same data as shown in A), but treatment with immune checkpoint inhibitors was included as an extra variable. * means significant differences in pairwise comparison after Benjamini Hochberg correction.
